# Channel-Specific Stimulus Sensitivity, Reliability, and Complementarity of Multimodal Wearable Sensing During Short-Form Influencer Video Exposure

**DOI:** 10.3390/s26196354

**Published:** 2026-10-08

**Authors:** Carolina Del-Valle-Soto, Violeta Corona, Regina Gómez Padilla, Fátima Sofía Walls Fernández, Juan-Carlos López-Pimentel, Carlos Mex-Perera, Paolo Visconti, José Varela-Aldás

**Affiliations:** 1 Facultad de Ingeniería, Universidad Panamericana, Álvaro del Portillo 49, Zapopan 45010, Jalisco, Mexico; cvalle@up.edu.mx (C.D.-V.-S.); 0262761@up.edu.mx (R.G.P.); 0266882@up.edu.mx (F.S.W.F.); clopezp@up.edu.mx (J.-C.L.-P.); 2 Facultad de Ciencias Económicas y Empresariales, Universidad Panamericana, Álvaro del Portillo 49, Zapopan 45010, Jalisco, Mexico; 3 College of Engineering Technology, Rochester Institute of Technology, One Lomb Memorial Drive, Rochester, NY 14623, USA; jmpiee@rit.edu; 4 Department of Innovation Engineering, University of Salento, 73100 Lecce, Italy; paolo.visconti@unisalento.it; 5 Centro de Investigaciones de Ciencias Humanas y de la Educación (CICHE), Universidad Tecnológica Indoamérica, Ambato 180103, Ecuador; josevarela@uti.edu.ec

**Keywords:** multimodal sensing, sensor fusion, electrodermal activity, photoplethysmography, eye tracking, facial expression analysis, variance components, measurement reliability, inter-subject correlation, short-form video

## Abstract

Multimodal wearable sensing is increasingly used to quantify audience engagement with short-form video, yet it remains unclear which sensing channels actually carry stimulus-driven information at the exposure durations typical of this format. We recorded electrodermal activity, photoplethysmographic pulse, triaxial acceleration, video-based eye tracking, and automated facial expression analysis from 50 young adults while they viewed 20 short-form influencer clips (5.6–19.3 s) presented in individually randomized order, yielding 1000 participant × clip trials and 12 primary indices spanning four modalities. Using crossed random-effects variance partitioning, split-half reliability with Spearman–Brown projection, permutation-based multivariate testing, participant-wise decoding, and inter-subject correlation, we show that stimulus-driven variance is strongly channel-specific: it reaches 13.7% for fixation rate and 10.2% for facial engagement but only 0.11% for pulse rate and 0.23% for RMSSD. Clip-level estimates were highly reliable for facial and oculomotor indices (r=0.82–0.94) and poor for autonomic indices (r=0.34–0.58), which would require 134–370 participants to reach ρ=0.80. Between-modality dependence was almost six times weaker than within-modality dependence, and five components were retained by parallel analysis. Oculomotor and facial features jointly identified individual clips at 16.8% balanced accuracy against a 5% chance level, whereas electrodermal and pulse features, taken separately, did not exceed chance. Electrodermal responses were nonetheless stimulus-locked in 18 of 20 clips. A per-second re-analysis of nine channels showed that the ordering partly reverses for within-clip dynamics: tonic electrodermal and pupil time courses were reliable across viewers ($\rho_{50}=0.88$ and 0.93), facial time courses were not ($\rho_{50}\leq 0.69$), and pulse rate was unreliable under both treatments. We derive explicit design guidance for sensor selection and sample size in short-exposure multimodal engagement studies.

## 1. Introduction

Short-form video has become the dominant vehicle of digital persuasion. Clips lasting between five and thirty seconds, distributed through creator-centric platforms and mediated by social media influencers, now account for a substantial share of the attention that audiences allocate to commercial and semi-commercial content [1,2]. The economic weight of this format has produced a corresponding methodological demand: advertisers, content producers, and researchers increasingly want objective, continuous, and low-burden measurements of how audiences respond, rather than relying exclusively on retrospective self-report, which is vulnerable to demand characteristics, memory distortion, and the well-documented dissociation between what viewers say and what they do [3,4].

Wearable and camera-based sensing offers an attractive answer. Contemporary research platforms synchronize electrodermal activity (EDA), photoplethysmography (PPG) or electrocardiography, accelerometry, video-based eye tracking, and automated facial expression analysis (FEA) into a single timeline, and expose vendor-computed indices, such as skin conductance response (SCR) rate, heart rate variability, fixation and saccade metrics, and action-unit intensities, that can be aligned to arbitrary stimulus intervals [5,6]. The implicit premise of this pipeline is that each additional sensor contributes incremental, stimulus-relevant information, so that fusing modalities yields a richer picture of engagement than any single channel could provide [7,8,9].

Recent work has carried this premise to new sensor types and fusion schemes. Bao et al. combined a polymer optical fiber (POF) cardiorespiratory sensor, which derives cardiac and respiratory signals from thoracic expansion, with a thermal imaging sensor of facial temperature, and showed that fusing features from the two modalities gave higher recognition accuracy than either modality alone [10]. Ensemble deep-learning models trained on multimodal wearable physiology [11], public datasets that pair facial expression with physiological recordings [12], and recent reviews of multimodal emotion recognition [13] follow the same logic: more channels, fused at the feature or decision level, should recognize affect better than any one channel. These studies evaluate fusion almost exclusively by classification accuracy over induction clips lasting tens of seconds to minutes. They rarely report how much of each channel’s variance is actually driven by the stimulus, or how reliable its stimulus-level estimates are, although these two quantities decide whether fusion can help at all when exposures are as short as a single short-form clip.

This premise deserves scrutiny at the specific temporal scale of short-form video. The physiological systems being sampled operate on markedly different time constants. The electrodermal response to a discrete event peaks one to five seconds after onset and returns to baseline over several more seconds [14,15]. Frequency-domain heart rate variability indices are formally defined over recording windows of at least two minutes, and even time-domain indices such as RMSSD become unstable in very short segments [16,17]. Oculomotor events, in contrast, occur several times per second, and facial action units are sampled at video frame rates [18,19]. A ten-second clip therefore provides hundreds of oculomotor and facial observations, roughly ten cardiac cycles, and frequently zero electrodermal responses. Whether the resulting indices are equally informative about the content being viewed is an empirical question that is rarely asked, because studies typically report only whether a given index differs significantly between conditions, without quantifying how much of its total variance is actually attributable to the stimulus.

A second, closely related issue is measurement reliability. In stimulus-set designs, the quantity of scientific interest is usually the clip-level mean, that is, the response that a given piece of content elicits, averaged across viewers. The precision of that quantity depends jointly on the number of viewers and on the proportion of trial-level variance that is stimulus-driven rather than person-driven or residual. When between-person variance dominates, as is characteristic of cardiovascular measures, clip-level means can be almost entirely noise even when the underlying trial-level data look well behaved [20,21]. Because reliability places a ceiling on any subsequent correlation, ranking, or predictive claim, quantifying it is a prerequisite for interpreting effect sizes in this literature.

A third issue concerns statistical inference over stimulus sets. Content is frequently organized into a small number of a priori categories, and inferential tests then treat individual trials as independent replicates. This is the classic language-as-fixed-effect fallacy: with five clips per category, the effective sample size for a category contrast is five, not the number of trials, and ignoring stimulus as a random factor inflates Type I error substantially [22,23,24]. Crossed random-effects models and stimulus-level permutation procedures address this directly and are now standard in adjacent fields, but remain uncommon in applied sensing studies.

The present work addresses these three issues in a single, fully instrumented dataset. Fifty young adults viewed twenty short-form influencer clips, organized a priori by the production team into four content blocks, while EDA, PPG, triaxial acceleration, gaze, and facial expression were recorded synchronously. The design is deliberately conventional and mirrors what a commercial or academic neuromarketing laboratory would actually run; the analysis is not. We ask, for each of twelve primary indices spanning four modalities, the following: (i) how much of its trial-level variance is attributable to the stimulus, once participant identity is modeled as a crossed random factor; (ii) how reliable the resulting clip-level estimates are, and how many participants would be required to reach a conventional reliability criterion; (iii) whether the a priori content blocks explain response variance beyond clip-level idiosyncrasy, tested with stimulus as the unit of inference; (iv) whether the modalities are statistically redundant or complementary; and (v) whether multimodal fusion permits identification of the individual clip a participant is watching, using participant-wise cross-validation and permutation-based null distributions.

Our contribution is therefore not another demonstration that physiology tracks media content, but a quantitative characterization of wherethe stimulus-driven signal resides in a multimodal sensing stack at short exposure durations, expressed in units that can be used directly to design subsequent studies: variance partition coefficients, reliability coefficients, required sample sizes, and decoding accuracies against explicit nulls. The five formal hypotheses tested are stated in Section 2.6. Section 2 describes the participants, instrumentation, feature extraction, quality control, and the statistical framework. Section 3 reports the results, Section 4 interprets them against the existing literature and states the limitations, and Section 5 summarizes the design implications.

## 2. Materials and Methods

### 2.1. Participants

Fifty young adults (26 male, 24 female; mean age 20.44 years, SD=1.43, range 18–24) were recruited from an undergraduate population and completed the full protocol. All participants had normal or corrected-to-normal vision compatible with remote eye tracking, reported no cardiovascular or dermatological conditions affecting the recorded signals, and were asked to refrain from caffeine in the two hours preceding the session. Data were collected in a single laboratory room under constant artificial illumination and controlled ambient temperature, with one participant tested at a time.

The study was conducted in accordance with the Declaration of Helsinki and approved by the institutional ethics committee (protocol code and date of approval are reported in the Institutional Review Board Statement). Written informed consent was obtained from every participant prior to instrumentation, including explicit consent for facial video recording and for the use of anonymized derived data. All records were pseudonymized at the point of analysis: participant names present in the acquisition export were replaced by opaque identifiers (P01–P50), and no facial imagery, raw videos, or directly identifying attributes are reported or released.

### 2.2. Stimuli and Experimental Design

The stimulus set comprised twenty short-form influencer video clips organized by the production team into four content blocks of five clips each, denoted as A, B, C, and D. Clip durations ranged from 5.57 to 19.33 s (mean 12.73 s, SD=3.69), with block means of 10.56 s (A), 11.89 s (B), 16.77 s (C), and 11.70 s (D); the full inventory is given in Section 3.1. Because duration is unevenly distributed across blocks, it was retained as a covariate in every block-level model and additionally removed by residualization in a control analysis of the decoding results (Section 2.7.6).

The four blocks were defined a priori by the production team when the stimulus set was assembled, before any participant was tested, and block membership was not revised after the responses had been inspected. The block labels are therefore a pre-specified grouping, not a partition derived from the data, and H3 is a confirmatory test of that grouping. The data-driven clustering reported in Section 3.4 was run afterwards and only to assess agreement with the pre-specified labels.

Each participant viewed all twenty clips, so the design is fully within-subjects and completely crossed: every participant × clip cell is observed exactly once, giving 50×20=1000 viewing trials. Presentation order was randomized independently for each participant, and the fifty realized sequences were mutually distinct, which decorrelates content from serial position and permits the estimation of within-session habituation as a separate effect. Clips were presented in full-screen mode with no interstimulus task, and no behavioral response was required during viewing, so that the recorded signals reflect passive exposure rather than task execution.

### 2.3. Instrumentation and Synchronization

Acquisition was performed on the iMotions multimodal research platform (version 10; iMotions A/S, Copenhagen, Denmark), which timestamps all incoming streams against a common clock and exports stimulus-aligned intervals. Four sensing subsystems were used.

**Electrodermal activity.** EDA was recorded with a Shimmer3 GSR+ unit (Shimmer Research Ltd., Dublin, Ireland) at 128 Hz using two Ag/AgCl electrodes on the non-dominant hand. The platform exports skin conductance in microsiemens after low-pass filtering, together with detected SCRs and a per-interval signal quality report.

**Cardiac activity.** Pulse was recorded with the optical photoplethysmographic channel of the same Shimmer3 GSR+ unit at 128 Hz. It is important to note that although the vendor pipeline labels the derived indices as “ECG metrics”, the underlying signal is photoplethysmographic; the resulting quantities are therefore pulse rate and pulse rate variability, not electrocardiographically derived heart rate variability. PPG-derived variability is a well-validated surrogate for short-term vagal indices at rest but is more sensitive to motion and peripheral vasomotion than the electrocardiogram [25,26], and we adopt the pulse terminology throughout.

**Body motion.** The triaxial accelerometer embedded in the Shimmer3 unit was recorded concurrently and used exclusively as a motion-artifact covariate. Movement occupied a mean of 1.74% of trial time, confirming that participants were largely still.

**Gaze.** Binocular gaze was recorded with a Smart Eye AI-X remote eye tracker (Smart Eye AB, Gothenburg, Sweden) at 60 Hz following a per-participant calibration whose outcome was graded by the platform as Excellent, Good, or Poor.

**Facial behavior.** Facial video was captured by webcam at 30 frames per second and processed with the AFFDEX 2.0 engine (Affectiva, Boston, MA, USA), which returns frame-wise likelihood scores for basic emotions, composite indices (engagement, valence, attention), and individual facial actions [27,28].

**Figure 1:**
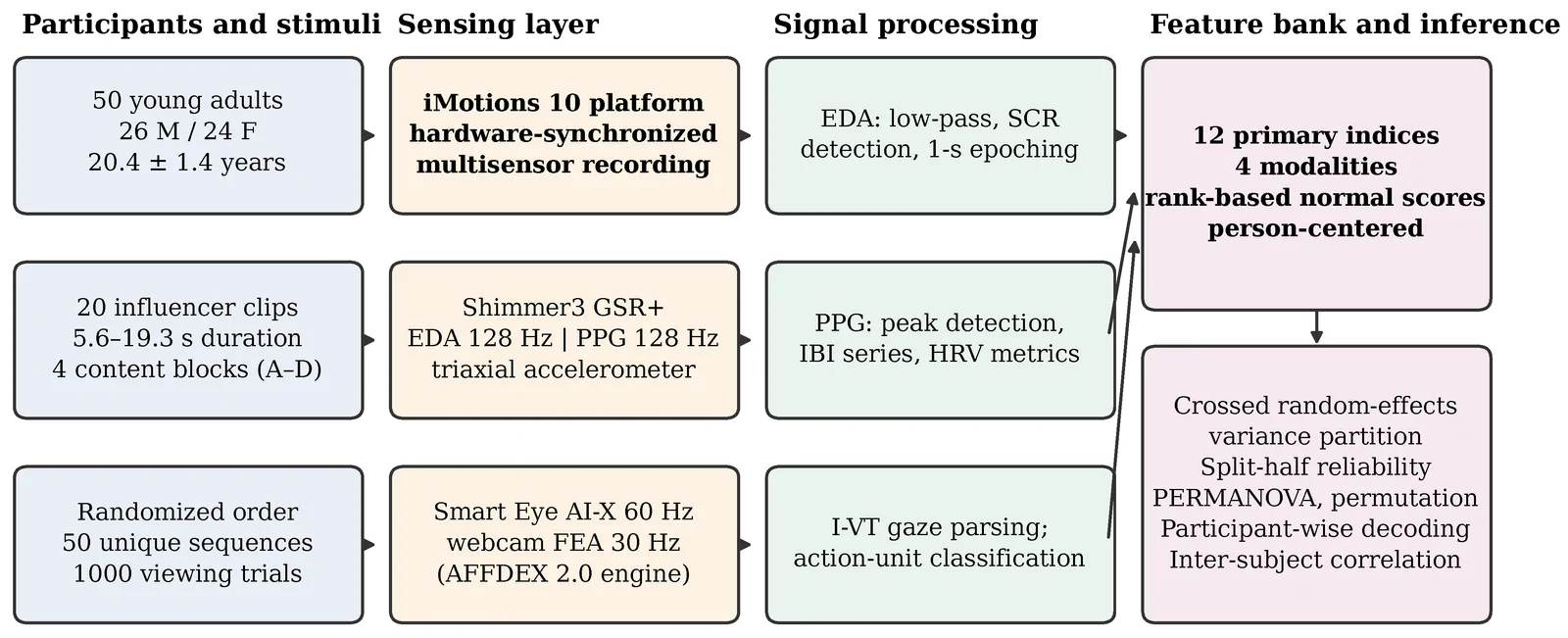
Overview of the study. Fifty participants viewed twenty short-form influencer clips in individually randomized order while five sensing channels were recorded synchronously. Vendor signal-processing flows produced event-level and interval-level outputs, from which twelve primary indices spanning four modalities were derived for each of the 1000 participant × clip trials. All inference was performed on rank-based normal scores with participant and stimulus modeled as crossed random factors.

Figure 1 summarizes the participants, the sensing layer, the signal-processing layer, and the statistical layer of the study.

### 2.4. Signal Processing and Feature Extraction

All features are defined at the level of a single participant × clip trial and, where the underlying quantity is a count, are expressed as rates in order to remove the mechanical dependence on clip duration.

**Electrodermal indices.** Two indices were retained. *SCR rate* is the number of detected skin conductance responses per minute of clip exposure. *SCL drift* is the ordinary-least-squares slope of the tonic skin conductance level across consecutive one-second epochs of the clip, expressed in μS s^−1^; it captures the direction and rate of tonic arousal change within the exposure, a quantity that phasic peak counts do not represent. Across the corpus, 587 SCRs were detected and the mean tonic level was 2.41 μS (SD=3.94).

**Cardiac indices.** *Pulse rate* is the mean instantaneous rate in beats per minute over the interval, and *log RMSSD* is the natural logarithm of the root mean square of successive interbeat-interval differences. Frequency-domain indices were computed by the platform but returned as missing for essentially all trials, as expected given that the shortest recommended window for low-frequency power exceeds the longest clip in the set by an order of magnitude [16]; they are therefore not analyzed.

**Oculomotor indices.** Gaze samples were parsed into fixations and saccades by the platform’s velocity-threshold identification algorithm [29], producing 21,634 fixations and 29,551 saccades in total. Six indices were derived per trial: *fixation rate* (fixations per minute), *median fixation duration* (ms), *gaze dispersion*, defined as $\sqrt{s_{x}^{2}+s_{y}^{2}}$, where $s_{x}$ and $s_{y}$ are the standard deviations of fixation centroid coordinates in pixels, *mean saccade amplitude* (degrees), *scanpath rate*, defined as the summed saccade amplitude divided by clip duration (deg s^−1^), and *blink rate* (blinks per minute) obtained from the eye tracker’s blink detector, with 3998 blinks detected in total. Median fixation duration was retained as a primary index even though it is partly reciprocal to fixation rate. The two quantities are not arithmetically redundant: fixation rate also depends on the time spent in saccades, blinks, and tracking loss, whereas the median duration describes how long gaze dwells once it lands and is insensitive to the few very long fixations that dominate the mean. It is also the oculomotor index most often interpreted as a marker of processing depth [18], so omitting it would have made the oculomotor set less comparable with prior work. Because the two indices were in fact strongly related in our data (Section 3.5), we additionally report a variant of fixation duration residualized on fixation rate (Section 3.9).

**Facial indices.** Two composite indices were retained: mean *facial engagement* and mean *facial valence* over the clip, both expressed on the engine’s percentage scale. Thresholded time-percentage variants and the full set of discrete emotion and action-unit channels were extracted and are reported descriptively, but were not entered into the primary inferential set in order to control multiplicity.

**Pupil diameter.** The eye tracker also reports pupil diameter for each eye in millimeters at 60 Hz. Invalid samples were discarded, the two eyes were averaged, and the trial mean was computed when at least 50% of the samples were valid, which was the case for 999 of the 1000 trials. Pupil diameter was not part of the pre-specified set and is dominated by the pupillary light reflex [30,31]; because frame-wise screen luminance was not recorded, luminance-driven and arousal-driven components cannot be separated in these data. We therefore analyze it as a supplementary index outside the primary family of twelve, both as a trial-level scalar and as a time series (Section 3.8), and interpret it as a test of response bandwidth rather than as an arousal measure.

The twelve primary indices, their modality assignment, and their descriptive statistics are given in Table 1.

### 2.5. Data Quality Control

**Figure 2:**
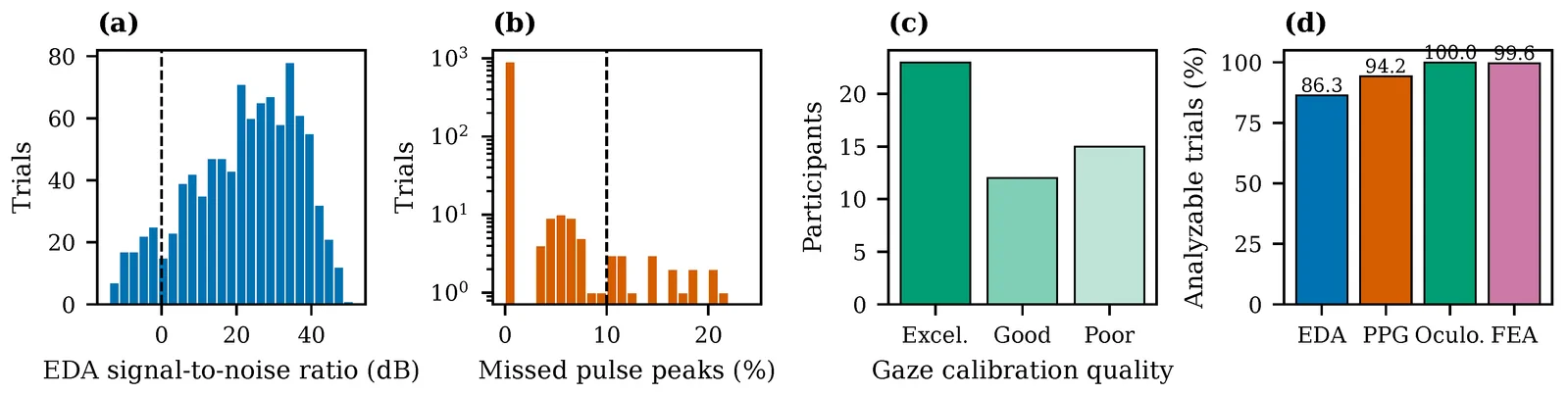
Signal quality of the multimodal corpus. (**a**) Distribution of electrodermal signal-to-noise ratio across trials; the dashed line marks the 0 dB inclusion threshold. (**b**) Percentage of missed pulse peaks per trial on a logarithmic count axis; the dashed line marks the 10% exclusion threshold. (**c**) Eye-tracker calibration grade per participant. (**d**) Percentage of the 1000 trials analyzable in each modality after quality control. Excel., Excellent; EDA, electrodermal activity; PPG, photoplethysmography; Oculo., oculomotor; FEA, facial expression analysis.

Quality control was applied before, and independently of, any hypothesis test. Two participants provided no Shimmer stream at all owing to a device failure, removing 40 trials from all autonomic analyses. For the remaining records, an electrodermal trial was declared analyzable if its signal-to-noise ratio exceeded 0 dB and less than 10% of the interval was missing, and a cardiac trial was declared analyzable if fewer than 10% of expected pulse peaks were missed. This yielded 863 analyzable electrodermal trials (86.3%) and 942 analyzable cardiac trials (94.2%); oculomotor features were available for all 1000 trials and facial features for 996. The median electrodermal signal-to-noise ratio was 23.5 dB (interquartile range 12.0–33.4) and the 95th percentile of missed pulse peaks was 4.5%. Gaze calibration was graded Excellent for 23 participants, Good for 12, and Poor for 15; because the last group is non-negligible, all variance-partition results were recomputed with those participants excluded as a robustness check (Section 3.9). One participant’s export contained a duplicated record set in which physiological and facial channels were split across two rows per clip; these were collapsed by non-missing coalescing, restoring exactly one record per participant × clip cell. Figure 2 summarizes signal quality.

### 2.6. Hypotheses

**Hypothesis 1**  (Channel-specific stimulus sensitivity)**.** *The proportion of trial-level variance attributable to the stimulus is greater than zero but differs systematically across sensing channels, being larger for channels whose effective sampling rate is high relative to clip duration* (*oculomotor, facial*)* than for channels whose response dynamics approach or exceed clip duration* (*electrodermal, cardiac*).

**Hypothesis 2**  (Reliability of clip-level estimates)**.** *The reliability of clip-level response estimates obtained from a sample of fifty viewers is sufficient for measurement-grade use* (ρ≥0.80)* for high-rate channels only, and the number of viewers required to reach that criterion differs across channels by an order of magnitude.*

**Hypothesis 3**  (A priori content structure)**.** *The four a priori content blocks explain multimodal response variance beyond the idiosyncratic contribution of individual clips, when stimulus is treated as the unit of inference.*

**Hypothesis 4**  (Modality complementarity)**.** *The sensing channels are not statistically redundant: dependence between modalities is substantially weaker than dependence within modalities, and the multimodal feature space requires more than one latent dimension.*

**Hypothesis 5**  (Multimodal decodability)**.** *Single-trial multimodal features permit above-chance identification of the individual clip being viewed under participant-wise cross-validation, and fusion of complementary modalities outperforms every constituent modality alone.*

### 2.7. Statistical Framework

#### 2.7.1. Normalization

Several indices are strongly skewed or zero-inflated (for example, SCR counts are zero in the majority of short trials, and facial engagement is bounded and highly leptokurtic). Rather than relying on parametric assumptions that these distributions violate, every primary index was converted to a rank-based inverse normal score using the Blom transform,

(1) $$z_{i}=\Phi^{-1}\;\left(\frac{r_{i}-3/8}{n+1/4}\right),$$

where $r_{i}$ is the rank of observation *i* among the *n* non-missing values of that index and $\Phi^{-1}$ is the standard normal quantile function. Because the transform is strictly monotone, it preserves the rank structure of the data while stabilizing variance and rendering likelihood-based and permutation-based inference directly comparable. Untransformed values are reported in all descriptive tables. For analyses that target stimulus-level structure, scores were additionally person-centered,

(2) $${\tilde{z}}_{ij}=z_{ij}-\frac{1}{J}\sum_{j^{\prime }=1}^{J}z_{ij^{\prime }},$$

with *i* indexing participants and *j* clips, which removes stable individual differences without altering within-participant contrasts.

#### 2.7.2. Variance Partition with Crossed Random Effects

For each index we fitted the crossed random-effects model

(3) $$z_{ij}=\mu +u_{i}+v_{j}+\varepsilon_{ij},\;u_{i}∼N\left(0,\sigma_{u}^{2}\right),\;v_{j}∼N\left(0,\sigma_{v}^{2}\right),\;\varepsilon_{ij}∼N\left(0,\sigma_{\varepsilon }^{2}\right),$$

where $u_{i}$ is a participant random intercept, $v_{j}$ a stimulus random intercept, and the three random terms are mutually independent. Parameters were estimated by restricted maximum likelihood. The variance partition coefficient attributable to the stimulus is

(4) $${\mathrm{VPC}}_{\mathrm{stim}}=\frac{\sigma_{v}^{2}}{\sigma_{u}^{2}+\sigma_{v}^{2}+\sigma_{\varepsilon }^{2}},$$

with analogous definitions for the participant and residual components. The null hypothesis $\sigma_{v}^{2}=0$ was tested by a likelihood-ratio statistic computed on maximum-likelihood fits, using the mixture reference distribution $\frac{1}{2}\chi_{0}^{2}+\frac{1}{2}\chi_{1}^{2}$ appropriate for a variance component on the boundary of the parameter space [32]. Because twelve indices were tested, *p*-values were controlled at a false discovery rate of 5% by the Benjamini–Hochberg procedure [33], and we report adjusted values as *q*.

#### 2.7.3. Reliability of Clip-Level Estimates

Clip-level reliability was estimated by repeated split-half resampling over participants. In each of 1000 iterations, participants were randomly partitioned into two halves of equal size, clip means of the person-centered scores were computed within each half, and the Pearson correlation $r_{1/2}$ between the two vectors of twenty clip means was recorded. The median of this distribution, together with its 2.5th and 97.5th percentiles, is reported. The reliability of the full sample follows from the Spearman–Brown prophecy formula,

(5) $$\rho_{k}=\frac{k\;\rho_{1}}{1+\left(k-1\right)\rho_{1}},$$

where $\rho_{1}$ is the reliability of a single observer, recovered from $r_{1/2}$ by inverting the same relation with k=n/2. Solving (5) for *k* gives the number of viewers required to reach a target reliability $\rho^{*}$,

(6) $$k^{*}=\frac{\rho^{*}\left(1-\rho_{1}\right)}{\rho_{1}\left(1-\rho^{*}\right)},$$

which we evaluate at $\rho^{*}=0.80$.

#### 2.7.4. Tests of the a Priori Content Structure

Block effects were tested in two complementary ways, both of which treat the stimulus as the unit of inference. First, the model of Equation (3) was extended with a fixed effect of block and covariates for standardized clip duration and standardized presentation position, and compared against the corresponding model without the block term by a likelihood-ratio test on three degrees of freedom; retaining $v_{j}$ in both models ensures that the contrast is evaluated against between-clip rather than between-trial variability, avoiding the language-as-fixed-effect fallacy [22,23]. Second, an exact-in-principle permutation test was applied to the twenty clip means, in which block labels were randomly reassigned to clips 9999 times and the one-way *F* statistic recomputed, yielding

(7) $$\hat{p}=\frac{1+\#\{b:F^{\left(b\right)}\geq F^{\mathrm{obs}}\}}{1+B}.$$

At the multivariate level, the twenty clips were represented by their twelve-dimensional standardized profiles and compared across blocks by permutational multivariate analysis of variance on Euclidean distances [34], with the pseudo-*F* statistic

(8) $$F=\frac{\left({\mathrm{SS}}_{T}-{\mathrm{SS}}_{W}\right)/\left(a-1\right)}{{\mathrm{SS}}_{W}/\left(N-a\right)},\;{\mathrm{SS}}_{W}=\sum_{g=1}^{a}\frac{1}{2n_{g}}\sum_{i,i^{\prime }\in g}d_{ii^{\prime }}^{2},$$

where ${\mathrm{SS}}_{T}$ is the total sum of squared interpoint distances divided by *N*, *a* the number of groups, $n_{g}$ the size of group *g*, and $d_{ii^{\prime }}$ the Euclidean distance between clip profiles; significance was obtained from 9999 label permutations. Finally, the clip profiles were clustered by Ward linkage into four groups and the agreement between the data-driven partition and the a priori blocks was quantified by the adjusted Rand index, with a permutation null obtained by shuffling the a priori labels.

#### 2.7.5. Dimensional Structure

Dependence among channels was summarized by Spearman correlations of person-centered scores with false-discovery-rate control across the 66 unique pairs. To quantify complementarity we compared the mean absolute correlation among pairs belonging to different modalities with the mean absolute correlation among pairs belonging to the same modality. Dimensionality was assessed by principal component analysis of the correlation matrix, with the number of retained components determined by parallel analysis against the 95th percentile of eigenvalues obtained from 500 random matrices of identical dimensions [35].

#### 2.7.6. Multimodal Decoding

To test H5 we asked whether the single-trial multimodal vector identifies the clip that produced it. Person-centered scores were used as features, missing values were imputed by the training-fold median, and multinomial logistic regression with $l_{2}$ penalization was fitted inside a nested cross-validation scheme in which both the outer five folds and the inner four folds were constructed by grouping on participant identity, so that no participant ever contributes to both the training and test sets. This is essential: with a within-subjects design, random splitting would allow the classifier to exploit participant-specific idiosyncrasies and would produce optimistically biased accuracies [36]. Performance is reported as balanced accuracy,

(9) $$\mathrm{BA}=\frac{1}{C}\sum_{c=1}^{C}\frac{{\mathrm{TP}}_{c}}{n_{c}},$$

whose chance level is 1/C, together with top-5 accuracy for the twenty-class problem. Statistical significance was established against an empirical null obtained by permuting class labels withineach participant and repeating the entire cross-validation procedure, which preserves the marginal design and provides a calibrated reference distribution [37]. Modality ablation compared the electrodermal, cardiac, oculomotor, and facial feature subsets and their unions under identical protocols. Because clip duration varies across blocks, the analysis was repeated on features linearly residualized on log duration.

#### 2.7.7. Inter-Subject Correlation of Electrodermal Dynamics

Although electrodermal indices carry little variance in the aggregate, this does not preclude time-locked responding. For each clip we assembled the participant × epoch matrix of one-second tonic conductance values, standardized each participant’s series within the trial, and computed the leave-one-out inter-subject correlation

(10) $$\mathrm{ISC}=\tanh\;\left(\frac{1}{n}\sum_{i=1}^{n}\mathrm{artanh}\;\mathrm{corr}\;\left(x_{i},\;\frac{1}{n-1}\sum_{i^{\prime }\neq i}x_{i^{\prime }}\right)\right),$$

following the approach introduced for neural time series [38,39]. The null distribution was generated by independently circularly shifting each participant’s series 1000 times, which preserves the autocorrelation structure of individual signals while destroying their temporal alignment; false discovery rate control was applied across the twenty clips.

Phasic timing was tested with an analogous procedure adapted to event data. For each clip, the onsets of SCRs detected in analyzable trials were binned into one-second intervals relative to media onset, and the concentration of onsets in time was summarized by the sum of squared bin counts of the resulting peri-stimulus histogram, which is largest when viewers respond in the same seconds. The null distribution was obtained by circularly shifting each participant’s onset train 2000 times, and false discovery rate control was applied across clips. Because SCRs are discrete and sparse, this test is less powerful than the tonic analysis, and we report it to establish whether phasic timing carries a usable stimulus-locked signal at these durations.

#### 2.7.8. Time-Resolved Comparison Across Channels

To test whether the channel ordering obtained for interval-averaged scalars also holds for time-locked dynamics, every channel was re-expressed as a per-second series on a common grid. For each participant × clip trial, samples were averaged within consecutive one-second bins from media onset, retaining the number of complete seconds available for every viewer of that clip (5 to 19 s; 242 s across the corpus). Nine channels were analyzed: tonic skin conductance (μS), pulse rate from the vendor interbeat-interval series (bpm), binocular pupil diameter (mm), gaze velocity (deg s^−1^), blink count, and the frame-wise AFFDEX outputs for facial engagement, facial valence, joy, and surprise, which were averaged over the roughly 30 frames of each second. The same trial-level quality criteria as in the main analysis were applied, gaps of up to two seconds were linearly interpolated, and series that were constant over the whole clip were excluded from standardization; for facial channels, the proportion of such flat series is itself reported as a measure of sparsity.

Each series was standardized within trial, which removes the trial mean and therefore any information already captured by the scalar indices. Three quantities were computed per channel. First, the leave-one-out ISC of Equation (10) was obtained per clip and tested against 1000 circular shifts, with false discovery rate control across clips. Second, each second was labeled as a responding second when the standardized value exceeded +0.5 SD, and agreement among viewers on these binary labels was quantified per clip by Fleiss’ κ, tested against 500 circular shifts. Third, the reliability of the clip-level time course was estimated exactly as for the scalars: in each of 1000 random splits of the participants, the group-mean time courses of the two halves were concatenated across the twenty clips and correlated, and the median correlation was projected to the full panel by Equation (5) and inverted by Equation (6). Because a response shape shared by all clips (for example, an orienting response at clip onset) would also inflate this correlation, the analysis was repeated after subtracting, within each half, the across-clip mean template expressed on normalized clip time. Finally, coupling between channels was quantified by correlating the concatenated group-mean time courses of each pair of channels, with a null obtained by circularly shifting one channel within each clip 1000 times and false discovery rate control across the 36 pairs.

#### 2.7.9. Software and Reproducibility

All analyses were performed in Python 3.13 (Python Software Foundation, Wilmington, DE, USA) using NumPy 2.4, pandas 3.0, SciPy 1.17, statsmodels 0.14, and scikit-learn 1.8, with figures produced in Matplotlib 3.10. Random seeds were fixed for every resampling procedure. Feature extraction operated on the platform’s exported event and interval tables; no raw video or raw waveform was re-processed, so the vendor algorithms are treated as the measurement instrument and are described as such.

Generative artificial intelligence was used to assist with code drafting for the analysis pipeline and with language editing of the manuscript. It was not used to generate, simulate, or alter data, nor to interpret results. All analyses were executed on the recorded data, and all authors verified the outputs reported here.

## 3. Results

### 3.1. Corpus and Descriptive Statistics

The analyzed corpus comprises 1000 complete participant × clip trials, exactly 50 per clip and 20 per participant. Table 1 reports the distribution of each primary index over the full corpus and by content block. Central tendencies are consistent with the literature for passive video viewing in young adults: mean pulse rate 78.1 bpm (SD=13.3), median fixation duration 386 ms, blink rate 18.6 min^−1^, and a low overall rate of electrodermal response (3.16 SCR min^−1^), with 587 responses detected across the corpus. Facial engagement and valence are heavily right-skewed, with medians near the engine’s floor and means inflated by a minority of strongly expressive trials, a distributional signature that motivates the rank-based normalization adopted here.

Table 2 gives the clip-level profile of the corpus in person-centered normal-score units, together with the electrodermal inter-subject correlation reported in Section 3.7. Clip-level values span roughly 1.4 standard deviations for fixation rate and facial engagement, indicating substantial content-driven modulation of those channels, and less than 0.25 standard deviations for pulse rate.

### 3.2. H1: Stimulus-Driven Variance Is Strongly Channel-Specific

**Figure 3:**
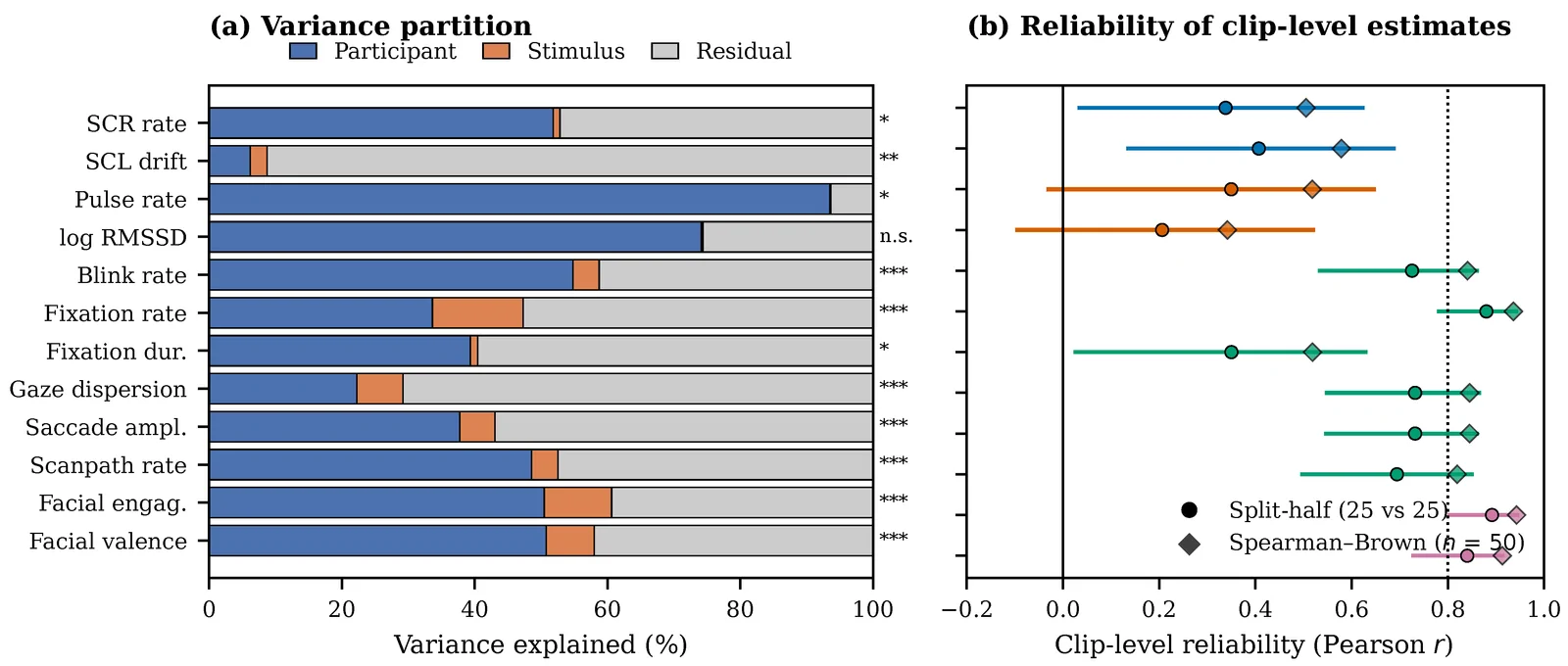
Where the stimulus-driven signal resides. (**a**) Variance partition of each primary index into participant, stimulus, and residual components, estimated by crossed random-effects models on rank-based normal scores; asterisks denote the significance of the stimulus variance component after false-discovery-rate control (* q<0.05, ** q<0.01, *** q<0.001). n.s., not significant; SCR, skin conductance response; SCL, skin conductance level; RMSSD, root mean square of successive differences; dur., duration; ampl., amplitude; engag., engagement. (**b**) Reliability of clip-level estimates: circles represent the median split-half correlation over 1000 random partitions of the 50 participants with 95% resampling intervals, and diamonds represent the Spearman–Brown projection to the full sample; the dotted line marks the conventional criterion ρ=0.80. The solid vertical line marks zero, and colors indicate the modality (blue, electrodermal; orange, pulse; green, oculomotor; pink, facial).

Table 3 and Figure 3a report the variance partition. The result is clear. Across every channel, between-participant variance is the largest identified component, ranging from 6.2% for electrodermal drift to 93.5% for pulse rate. The stimulus component, in contrast, varies by two orders of magnitude across channels: it reaches 13.65% for fixation rate, 10.15% for facial engagement, 7.22% for facial valence, and 6.95% for gaze dispersion, but falls to 1.01% for SCR rate, 0.23% for log RMSSD, and 0.11% for pulse rate.

Likelihood-ratio tests confirm that the stimulus component is significantly non-zero for ten of the twelve indices after false-discovery-rate control, the exceptions being log RMSSD (q=0.125) and, marginally significant but negligible in magnitude, pulse rate (${\mathrm{VPC}}_{\mathrm{stim}}=0.11\%$, q=0.025). H1 is therefore supported in its qualitative form (stimulus-driven variance exists in most channels) and, more importantly, in its quantitative form: the ordering of channels follows the ratio between the response bandwidth of the underlying physiological system and the duration of the exposure. Channels sampling events at rates of one per second or faster carry six to more than one hundred times more stimulus variance than channels whose characteristic response time is comparable to the clip itself.

Pupil diameter, analyzed as a supplementary index outside the primary family, had the largest participant component of any channel except pulse rate (85.8%), a stimulus component of 3.83% (LRT =242.7, p<0.001), and a clip-level split-half reliability of $r_{1/2}=0.91$ [0.82, 0.96] ($\rho_{50}=0.95$, $k^{*}=9$; Table 3). It was essentially unrelated to the twelve primary indices (|ρ|≤0.13). A fast autonomic channel can therefore resolve clips reliably at this exposure length; given that luminance was not controlled, however, this result speaks to response bandwidth and not to arousal.

### 3.3. H2: Clip-Level Reliability Separates Usable from Unusable Channels

The reliability analysis translates the variance partition into a directly actionable quantity. Split-half correlations of clip means (Table 3, Figure 3b) are high for facial engagement ($r_{1/2}=0.89$; $\rho_{50}=0.94$), fixation rate (0.88; 0.94), facial valence (0.84; 0.91), gaze dispersion and saccade amplitude (both 0.73; 0.85), blink rate (0.73; 0.84), and scanpath rate (0.69; 0.82). All seven exceed the conventional ρ=0.80 criterion at n=50, and four of them would reach it with fewer than 40 viewers (facial engagement with as few as 12).

**Figure 4:**
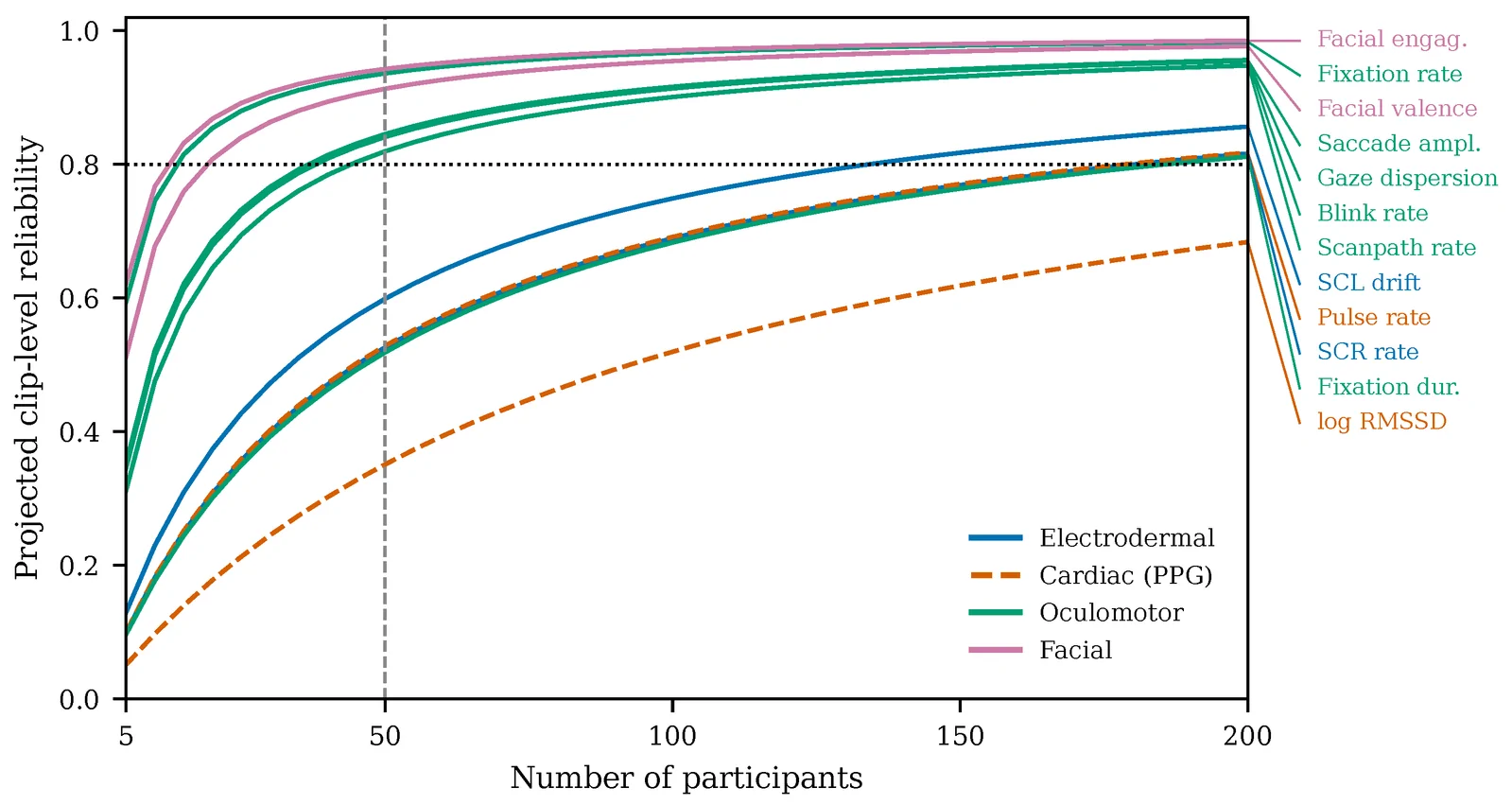
Projected clip-level reliability as a function of panel size, obtained by applying the Spearman–Brown prophecy formula to the single-observer reliability of each index. The vertical dashed line marks the panel size of the present study; the horizontal dotted line marks ρ=0.80. Facial and high-rate oculomotor indices cross the criterion below 50 viewers, whereas autonomic indices remain below it across the entire plotted range. PPG, photoplethysmography; SCR, skin conductance response; SCL, skin conductance level; RMSSD, root mean square of successive differences; dur., duration; ampl., amplitude; engag., engagement.

The remaining five indices fail decisively. SCR rate, SCL drift, pulse rate, and median fixation duration reach $\rho_{50}$ between 0.51 and 0.58, and log RMSSD only 0.34, with a 95% resampling interval for the split-half correlation that includes zero. Extrapolating with Equation (6), reaching ρ=0.80 would require 134 viewers for electrodermal drift, roughly 180 for SCR rate, pulse rate, and median fixation duration, and 370 for log RMSSD (Figure 4). H2 is therefore supported: measurement-grade clip-level estimation from a fifty-viewer panel is achievable for facial and most oculomotor channels and is not achievable for autonomic channels, with the required panel size differing by more than an order of magnitude across the sensing stack.

### 3.4. H3: The a Priori Content Blocks Explain Little Beyond Clip Identity

**Figure 5:**
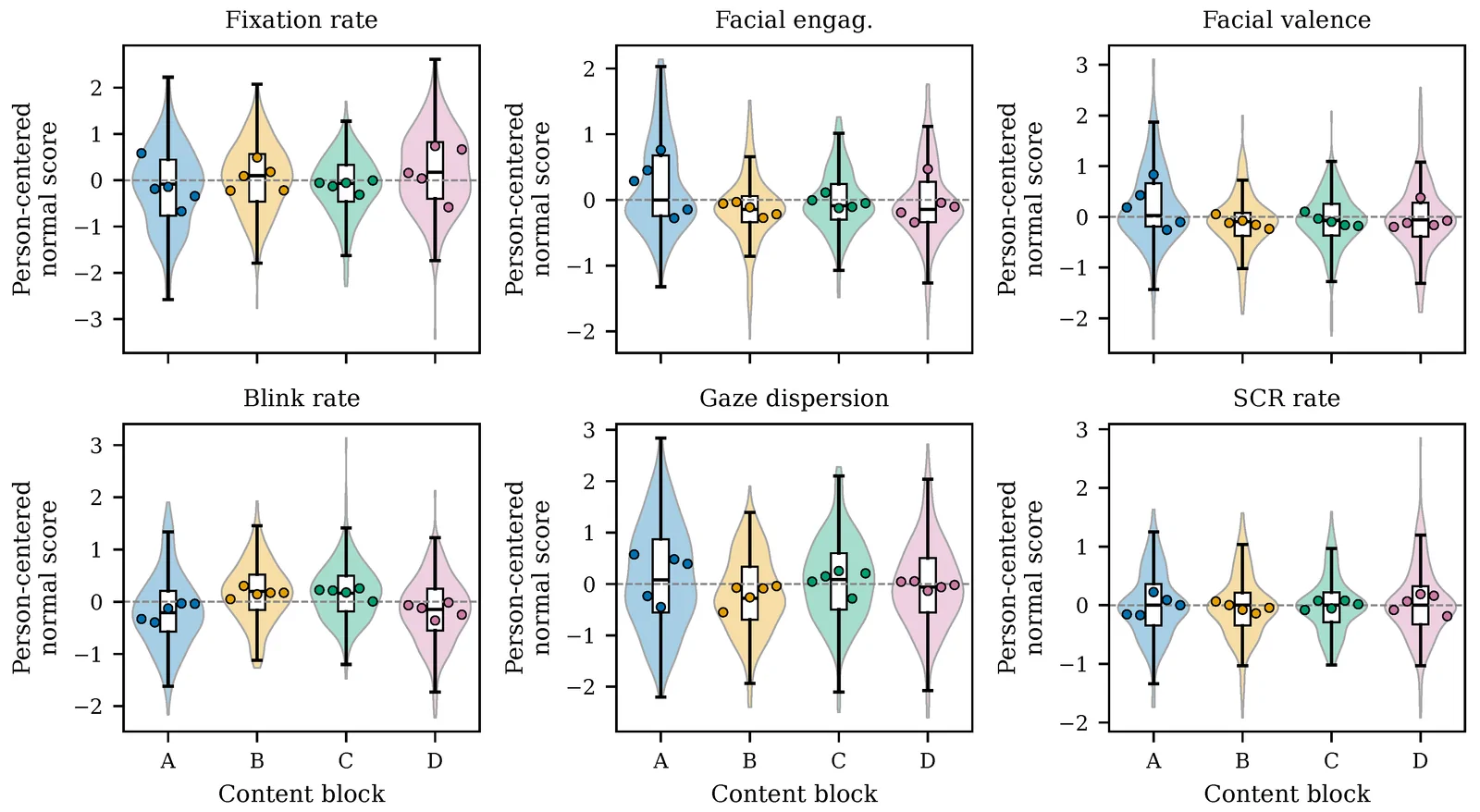
Distribution of six representative indices by content block. Violins and boxplots show person-centered normal scores across all trials; superimposed circles are the twenty clip-level means, which are the effective units of inference for a block contrast. The dispersion of clip means within each block is comparable to the dispersion between blocks for every index except blink rate.

Having established that individual clips modulate several channels reliably, we asked whether the four a priori content blocks capture that modulation. They largely do not. In the crossed random-effects models with duration and presentation position as covariates (Table 4; Figure 5), only blink rate shows a block effect surviving correction (LRT(3)=19.19, $p=2\times 10^{-4}$, q=0.003), with higher rates for blocks B and C (+0.18 normal-score units) than for A and D (−0.17 and −0.15). Facial engagement, facial valence, and saccade amplitude show numerically ordered means favoring block A but do not survive correction (q≥0.35). The stimulus-level permutation test reproduces this pattern exactly: blink rate is significant (F=12.06, $p_{\mathrm{perm}}=4\times 10^{-4}$, q=0.005) and no other index approaches significance.

**Figure 6:**
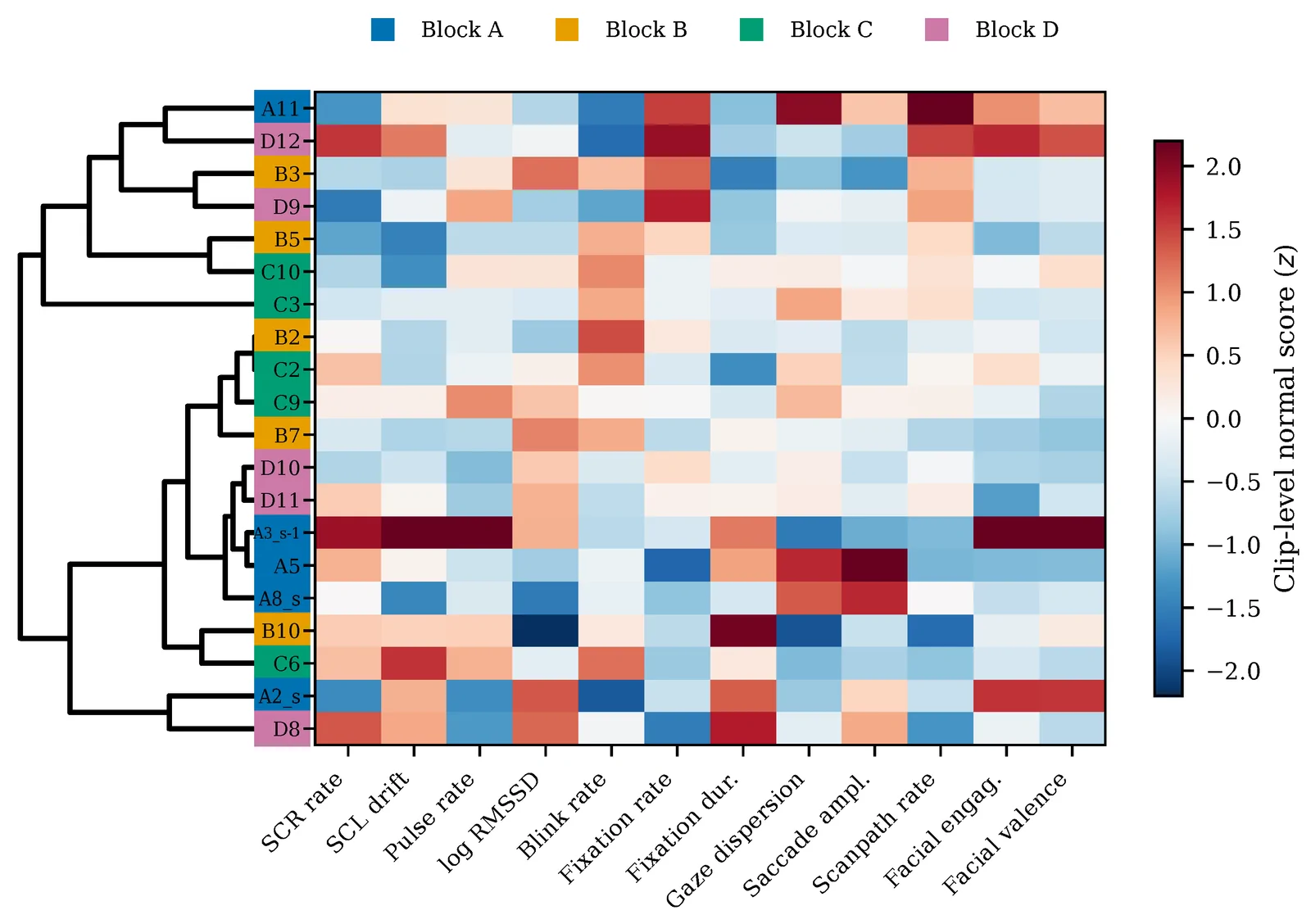
Clip-level multimodal profiles with Ward hierarchical clustering. Rows are the twenty clips, colored bars on the left indicate the a priori content block, and cells show the clip-level normal score of each index. The data-driven partition into four clusters agrees with the a priori blocks only at chance level (adjusted Rand index =0.075, p=0.148). SCR, skin conductance response; SCL, skin conductance level; RMSSD, root mean square of successive differences; dur., duration; ampl., amplitude; engag., engagement.

The multivariate test agrees. PERMANOVA on the twelve-dimensional clip profiles yields F=1.30 with $R^{2}=0.196$ and p=0.175: the block factor accounts for about a fifth of the between-clip dispersion, which is no more than expected from a random four-way partition of twenty points in twelve dimensions. Data-driven clustering confirms the misalignment. Ward clustering of the clip profiles into four groups agrees with the a priori blocks only at the level of chance (adjusted Rand index =0.075, p=0.148); the resulting dendrogram (Figure 6) groups clips across rather than within blocks, with the strongly expressive clips A3_s-1, A2_s, A11, and D12 forming a cluster that spans two blocks.

H3 is therefore rejected. The reliable content signal identified under H1 and H2 lives at the level of individual clips, not at the level of the four production-defined categories.

### 3.5. H4: Modalities Are Complementary, Not Redundant

**Figure 7:**
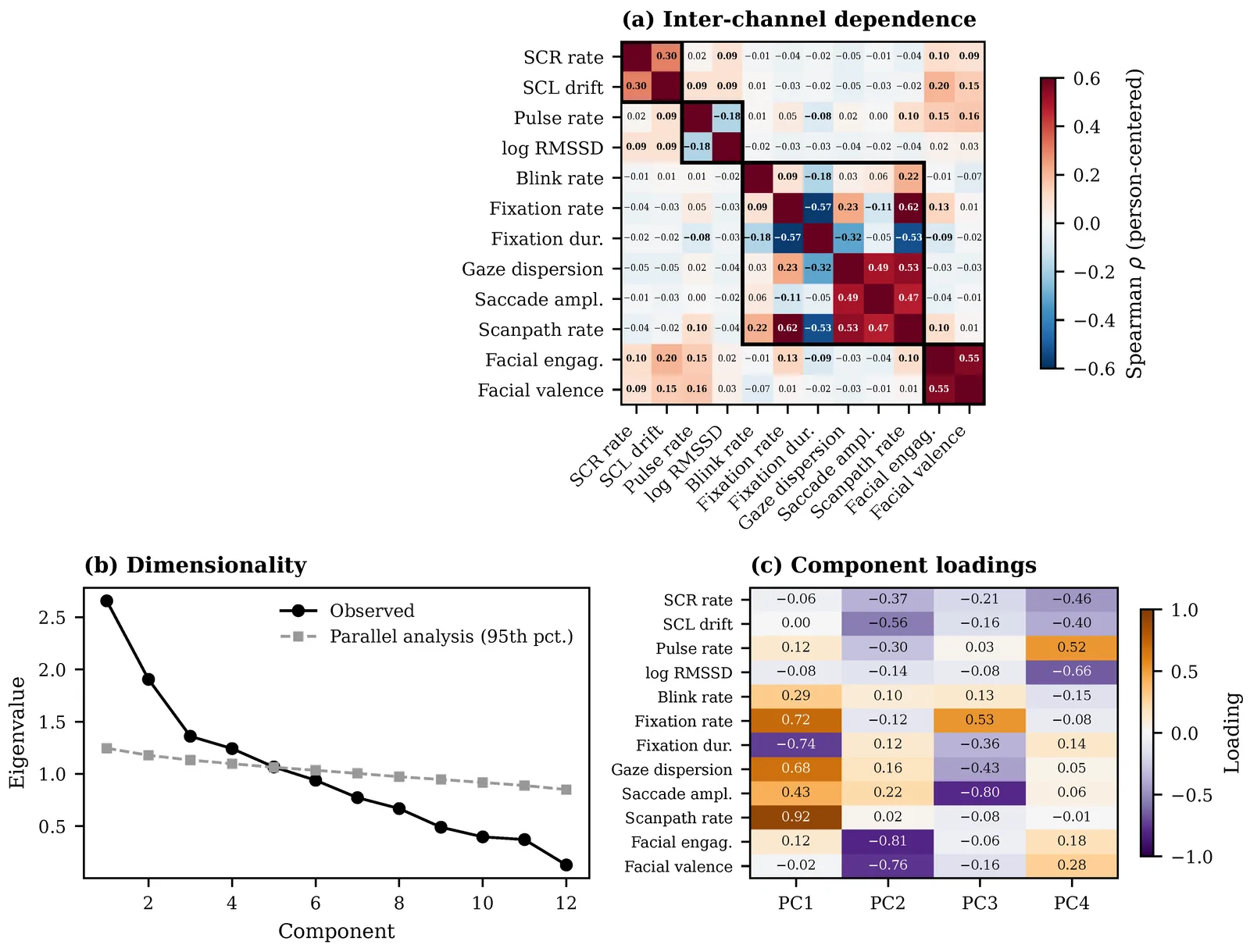
Dimensional structure of the multimodal response. (**a**) Spearman correlations among person-centered indices; black frames delimit modalities and values in bold mark associations surviving false-discovery-rate control across the 66 unique pairs. (**b**) Observed eigenvalues against the 95th percentile of eigenvalues from 500 random matrices of identical dimensions; five components are retained. (**c**) Loadings on the first four components. SCR, skin conductance response; SCL, skin conductance level; RMSSD, root mean square of successive differences; dur., duration; ampl., amplitude; engag., engagement; PC, principal component.

Figure 7a shows the Spearman correlation matrix of person-centered scores. Dependence is organized in a clear block-diagonal pattern along modality boundaries. Within modalities, associations are moderate to strong and physiologically interpretable: facial engagement and valence correlate at ρ=0.55; scanpath rate correlates with fixation rate at ρ=0.62 and with gaze dispersion at ρ=0.47; fixation rate and median fixation duration are strongly negatively related (ρ=−0.57), as expected from their reciprocal definition; and SCR rate correlates with SCL drift at ρ=0.30.

Between modalities, dependence essentially vanishes. The mean absolute correlation across modality boundaries is 0.052, compared with 0.307 within modalities, a ratio of 5.9. The largest cross-modality associations are small and involve facial and electrodermal channels (|ρ|≤0.20). Parallel analysis retains five principal components, which jointly explain 68.6% of the variance (22.1, 15.9, 11.3, 10.4, and 8.9%), and the loading pattern (Figure 7c) recovers interpretable and largely modality-aligned dimensions: a first component dominated by scanpath rate, fixation rate, gaze dispersion, and inverted fixation duration, describing visual exploration; a second dominated by facial engagement and valence with negative electrodermal contributions, describing expressive positivity; a third contrasting saccade amplitude with fixation rate, describing scanning granularity; and a fourth loading on cardiac indices.

H4 is thus supported. The four modalities are not measuring a single underlying construct that a single sensor could recover; they occupy nearly orthogonal subspaces of the response, which is precisely the condition under which sensor fusion is informative rather than merely redundant.

### 3.6. H5: Multimodal Fusion Identifies Individual Clips Above Chance

Under participant-wise nested cross-validation, the twelve-dimensional multimodal vector of a single trial identifies which of the twenty clips produced it with a balanced accuracy of 16.2%, against an empirical null of 5.02±0.76% (p=0.005; chance =5%) and a top-5 accuracy of 51.4%. Because clip duration is confounded with block, we repeated the analysis on features linearly residualized on log duration; accuracy fell only to 15.1% and remained significant (p=0.010), showing that decoding is not an artifact of exposure length. For the four-way block problem, balanced accuracy was 40.7% against a null of 25.0±1.6% (p=0.005), which is above chance but consistent with the univariate finding that blink rate, and to a lesser degree facial expressivity, carry weak block-level information.

**Figure 8:**
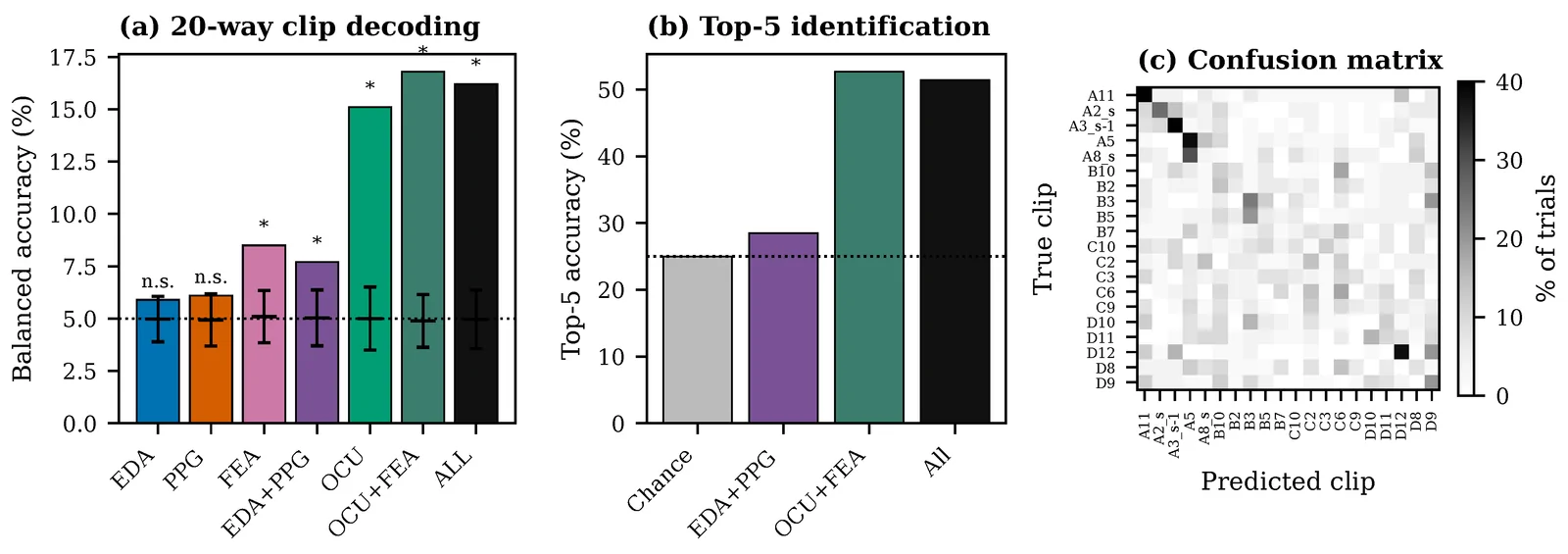
Multimodal decoding of clip identity. (**a**) Balanced accuracy by feature set; horizontal markers with error bars represent the mean and 95% range of the within-participant permutation null, and the dotted line marks the 5% chance level; *, p<0.05 against the permutation null; n.s., not significant. (**b**) Top-5 accuracy against a 25% chance level, marked by the dotted line. (**c**) Row-normalized confusion matrix of the full multimodal model. EDA, electrodermal activity; PPG, photoplethysmographic pulse; FEA, facial expression analysis; OCU, oculomotor.

Modality ablation (Table 5, Figure 8) closely reproduces the variance-partition ordering. Oculomotor features alone reach 15.1% and facial features alone 8.5%, both significantly above chance, whereas electrodermal features (5.9%, p=0.079) and pulse (PPG) features (6.1%, p=0.059) do not exceed chance, and their union reaches only 7.7%. The best-performing configuration is the fusion of oculomotor and facial features (16.8% balanced accuracy, 52.7% top-5), which exceeds each of its constituents and also exceeds the full twelve-feature model (16.2%), indicating that the autonomic features contribute noise rather than signal at this exposure duration.

H5 is therefore supported with an important qualification: fusion helps, but only fusion of the channels that individually carry stimulus variance. Indiscriminate concatenation of all available sensors slightly degraded performance.

### 3.7. Electrodermal Responses Are Time-Locked Even When Aggregate Indices Are Not

**Figure 9:**
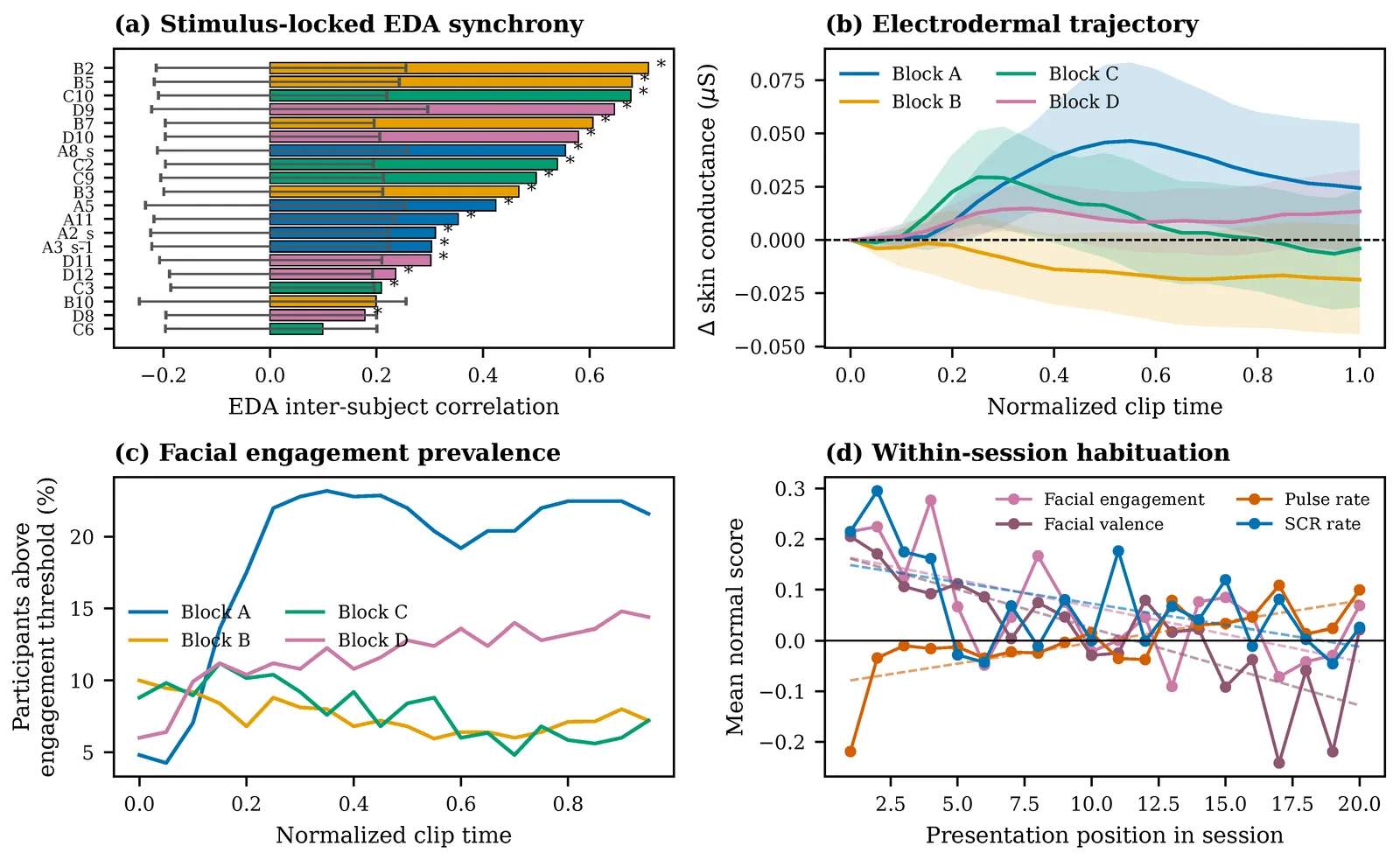
Temporal dynamics of the multimodal response. (**a**) Leave-one-out inter-subject correlation of tonic electrodermal activity per clip, colored by content block; horizontal bars represent the mean and 95% range of the circular-shift null, and asterisks mark clips significant after false-discovery-rate control. (**b**) Baseline-corrected electrodermal trajectories over normalized clip time, averaged within block, with 95% confidence ribbons; the dashed line marks zero change from baseline. (**c**) Percentage of viewers exceeding the facial engagement threshold over normalized clip time. (**d**) Mean normal scores as a function of presentation position in the session, with least-squares trends (dashed lines).

The failure of electrodermal indices in the aggregate analyses could mean either that skin conductance does not respond to this material or that it responds in a way that interval-averaged indices discard. The inter-subject correlation analysis discriminates between these alternatives. Tonic electrodermal trajectories were significantly synchronized across viewers in 18 of the 20 clips after false-discovery-rate control, with a median ISC of 0.446 and values as high as 0.71 (clip B2), 0.68 (B5, C10), and 0.65 (D9); only B10 (q=0.068) and C6 (q=0.176) failed to reach significance (Figure 9a). Circular-shift nulls were centered near zero in every case, confirming that the effect reflects temporal alignment rather than shared autocorrelation.

Phasic timing did not show the same synchrony. Across clips, a median of 18 of the 40–45 analyzable viewers produced at least one SCR, contributing on average 28.9 onsets per clip. Onsets were more concentrated in time than the circular-shift null nominally for three clips (A3_s-1, A8_s, and D12; p=0.007–0.044), but for none after false-discovery-rate control (all q≥0.14), and the Fisher combination across the twenty clips was only marginal (p=0.021). At these durations, discrete responses are too sparse to support a stable time-locked estimate, which is why the synchrony analysis was based on the tonic signal.

Electrodermal synchrony was, moreover, inversely related to the aggregate phasic index: clips eliciting more synchronized tonic trajectories elicited fewerdiscrete SCRs (ρ=−0.537, p=0.015), and ISC was unrelated to clip duration (ρ=−0.221, p=0.349) or to facial engagement (ρ=−0.167, p=0.482). Interval-averaged SCR counts therefore do not merely lack power; they index a partially distinct aspect of electrodermal response from the shared temporal envelope that the stimulus imposes on tonic arousal.

Two additional temporal findings complete the picture. Baseline-corrected electrodermal trajectories showed no systematic within-clip trend (t(49)=0.03, p=0.976), so the synchrony reflects clip-specific shapes rather than a common linear ramp (Figure 9b); a non-linear response shape shared by all clips is examined in Section 3.8. Across the session, however, engagement declined monotonically with presentation position: facial engagement (β=−0.071, q<0.001), facial valence (β=−0.093, q<0.001), SCR rate (β=−0.060, q=0.004), and log RMSSD (β=−0.082, q<0.001) all decreased, while pulse rate increased (β=+0.049, q<0.001) and scanpath rate increased (β=+0.051, q=0.039), a coherent habituation-plus-fatigue signature that is only estimable because presentation order was randomized (Figure 9d, Table A1).

### 3.8. Time-Resolved Comparison: The Channel Ordering Changes for Dynamics

**Figure 10:**
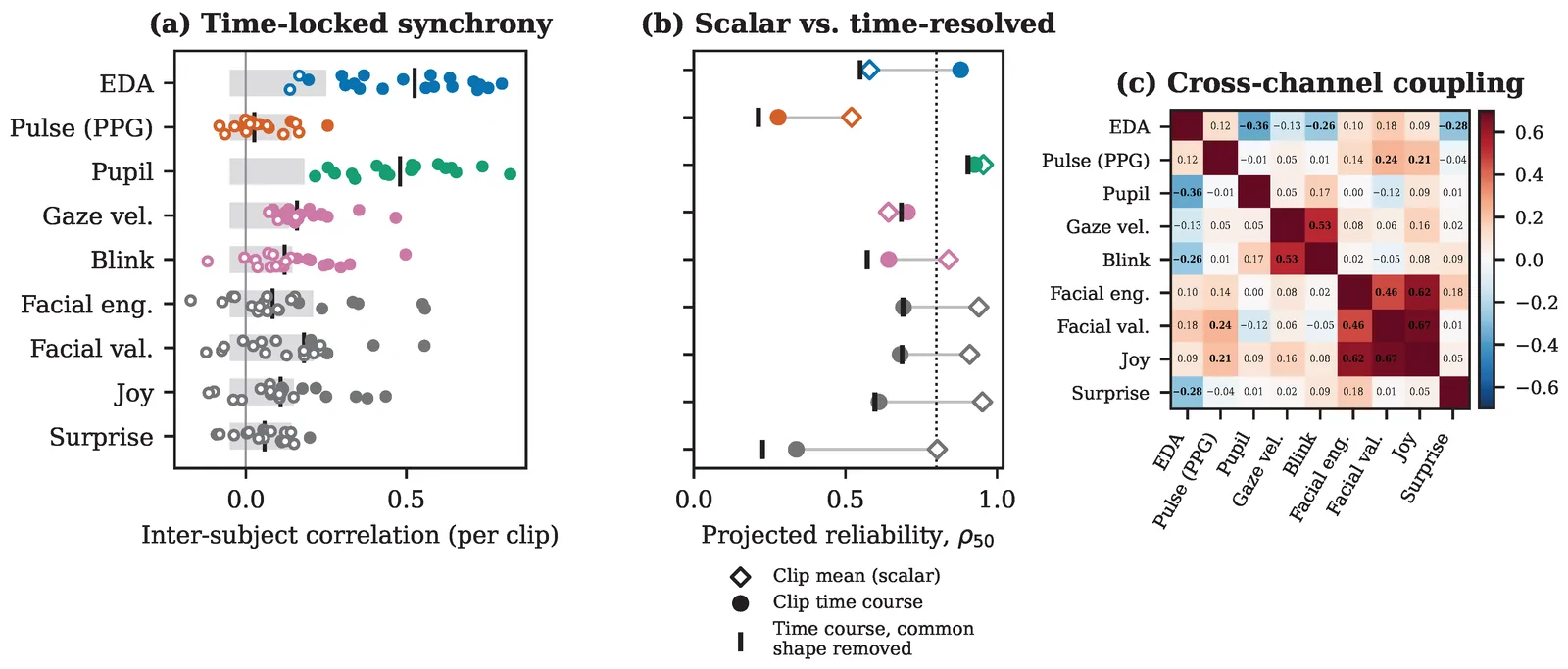
Time-resolved comparison across channels. (**a**) Leave-one-out inter-subject correlation of per-second series for each clip; filled circles are clips significant after false-discovery-rate control, open circles are not, vertical bars mark the median, and gray bands show the mean 97.5th percentile of the circular-shift null. (**b**) Projected clip-level reliability at 50 viewers for the clip mean (diamonds; values from Table 3 or computed identically) and for the clip time course (circles), with the time-course value after removal of the common across-clip shape (vertical ticks); the dotted line marks ρ=0.80. (**c**) Correlations between channels of the concatenated group-mean time courses; values in bold mark pairs surviving false-discovery-rate control across the 36 pairs. EDA, electrodermal activity; PPG, photoplethysmography; vel., velocity; eng., engagement; val., valence.

Table 6 and Figure 10 report the per-second analysis of Section 2.7.8. The ordering obtained for interval-averaged scalars does not carry over to within-clip dynamics. Tonic electrodermal activity and pupil diameter were the most strongly time-locked channels, with median ISCs of 0.53 and 0.48, significant synchrony in 18 and 20 of the 20 clips, and time-course reliabilities above the 0.80 criterion at n=50 ($\rho_{50}=0.88$, $k^{*}=27$, and $\rho_{50}=0.93$, $k^{*}=16$). Gaze velocity (median ISC 0.16, 17 clips) and blink count (0.12, 8 clips) were intermediate, with $\rho_{50}$ of 0.70 and 0.64. The facial channels, which produced the most reliable scalars, produced some of the least reliable time courses: facial engagement and valence were flat at the engine floor for the whole clip in 44% and 47% of trials, synchrony reached significance in only six clips each, and $\rho_{50}$ was 0.69 and 0.68 ($k^{*}\approx 90$). AFFDEX joy behaved similarly ($\rho_{50}=0.61$), and surprise barely tracked the stimulus ($\rho_{50}=0.34$). Pulse rate was unreliable under both treatments (median ISC 0.03, one clip, $\rho_{50}=0.28$, $k^{*}\approx 520$). The electrodermal ISC values obtained on this common grid are slightly higher than those of Section 3.7 (median 0.53 versus 0.45), because the grid is built from the sample-level conductance signal rather than from the vendor epochs, but the same 18 clips are significant.

The per-second labels give the same picture in terms of categorical agreement. Viewers agreed on which seconds were responding seconds beyond the circular-shift null in 17 clips for electrodermal activity and 18 for pupil diameter, but in only 1 to 8 clips for the oculomotor, facial, and pulse channels. Absolute agreement was modest for every channel (median Fleiss’ κ≤0.10), as expected when a binary label is imposed on a continuous signal, but its ordering followed that of the ISC.

Two qualifications apply. First, part of the electrodermal time-course reliability reflects a response shape common to all clips, most plausibly an orienting response to clip onset. When the across-clip template was removed, the split-half correlation for electrodermal activity fell from 0.76 to 0.34 ($\rho_{50}$ from 0.88 to 0.55), whereas $\rho_{50}$ was practically unchanged for pupil diameter (0.93 to 0.90), gaze velocity (0.70 to 0.68), and the facial channels. The clip-specific part of the electrodermal dynamics is therefore real but smaller than the raw ISC suggests. Second, the high reliability of the pupil time course cannot be attributed to arousal, because luminance changes within each clip drive the pupil directly [30,31].

Across channels, the group-level time courses were largely independent (Figure 10c). The couplings that survived false-discovery-rate control were within the facial modality (joy with engagement, r=0.62; joy with valence, 0.67; engagement with valence, 0.46) and within the oculomotor modality (gaze velocity with blink count, 0.53). The only cross-modal couplings were negative associations of electrodermal activity with pupil diameter (−0.36), blink count (−0.26), and surprise (−0.28), and weak positive associations of pulse rate with valence (0.24) and joy (0.21). The complementarity found for scalars under H4 thus also holds for time-locked dynamics.

Finally, the frame-wise AFFDEX emotion outputs are sparse. Of 377,741 analyzed facial frames, 7.5% had a likelihood of 50 or more (the vendor default threshold) for any of the seven basic emotions, and 98% of those were joy (7.3% of all frames). Surprise, anger, contempt, disgust, fear, and sadness each exceeded the threshold in 0.13% of frames or fewer. Thirteen of the 50 participants never produced a single supra-threshold frame, and only 18.5% of trials contained at least one supra-threshold second. A per-second categorical emotion label is therefore “neutral” for 92.6% of the corpus, which is why the frame-wise outputs were analyzed here as continuous likelihoods rather than as discrete labels.

### 3.9. Robustness and Covariate Checks

Three checks support the stability of the main results. First, excluding the fifteen participants whose gaze calibration was graded Poor changed the stimulus variance partition coefficients by at most 3.5 percentage points, and never reversed the ordering of channels; for fixation rate the exclusion *increased* ${\mathrm{VPC}}_{\mathrm{stim}}$ from 13.65% to 17.13%, as expected if calibration error adds stimulus-independent noise (Table A2). Second, clip duration had no significant effect on any primary index after false-discovery-rate control (all q≥0.30), so the duration imbalance across blocks does not drive the block-level conclusions. Third, participant sex and age were unrelated to the primary indices after correction (all q≥0.28 and q≥0.48, respectively); the largest uncorrected effect, a higher SCR rate in male participants (β=0.44, p=0.008), did not survive correction (q=0.091). Full coefficients are given in Table A1.

Two further checks concern the Poor-calibration group and median fixation duration. The fifteen participants with Poor calibration did not differ from the other 35 in age (median 20 versus 21 years, Mann–Whitney p=0.45), facial engagement (p=0.70), facial valence (p=0.79), SCR rate (p=0.32), blink rate (p=1.00), fixation rate (p=0.07), gaze dispersion (p=0.51), body motion (p=0.49), or the proportion of valid pupil samples (p=0.85). Their mean pulse rate (72 versus 81 bpm, p=0.025) and mean pupil diameter (4.31 versus 4.86 mm, p=0.012) were nominally lower, but neither difference survived false-discovery-rate control across the eleven comparisons (q=0.14 for both). The group did differ in sex composition (12 of 15 male, versus 14 of 35; Fisher’s exact p=0.014). Because sex was unrelated to every primary index after correction and the exclusion of the group left the channel ordering unchanged, this imbalance does not affect the main conclusions, but it means that the calibration check in Table A2 is partly also a check on sex composition. Finally, median fixation duration and fixation rate were strongly related (ρ=−0.57). When fixation duration was residualized on fixation rate within each participant, the remaining component carried 5.3% stimulus variance relative to within-participant variance, compared with 1.8% for the raw index on the same scale (1.10% of total variance in Table 3), and its clip means had a split-half reliability of $r_{1/2}=0.60$ ($\rho_{50}=0.75$, $k^{*}=67$) compared with 0.35 ($\rho_{50}=0.52$) for the raw index. The part of fixation duration that is not explained by fixation rate is therefore more, not less, stimulus-specific than the raw index, and the reciprocal dependence does not account for its poor reliability.

## 4. Discussion

### 4.1. Principal Findings

This study asked where, in a conventional multimodal sensing stack, the stimulus-driven signal actually resides when the exposures are as short as those that dominate contemporary video consumption. Four results answer this question. First, stimulus-driven variance is present but strongly channel-specific, spanning two orders of magnitude from 13.7% for fixation rate to 0.11% for pulse rate. Second, this translates into a sharp reliability dichotomy: facial and high-rate oculomotor indices yield measurement-grade clip-level estimates from a fifty-viewer panel, whereas autonomic indices would require 134 to 370 viewers for the same precision. Third, the modalities are close to statistically orthogonal, with between-modality dependence six times weaker than within-modality dependence and five retained components, so the multimodal stack is genuinely multidimensional. Fourth, fusion of the informative channels decodes individual clip identity at more than three times chance, while the addition of autonomic channels degrades rather than improves performance.

The time-resolved analysis adds an important qualification to the first two results. When channels are compared on their within-clip dynamics instead of on interval averages, tonic electrodermal activity and pupil diameter become the most reliable channels and facial expression drops to an intermediate position, whereas pulse rate remains unreliable either way. Which channel is “best” therefore depends on the summary statistic, not only on the sensor. A fifth result is negative and, in our view, equally instructive: the four a priori content blocks explain almost nothing beyond the identity of individual clips. This holds univariately, multivariately, and in a data-driven clustering that agrees with the a priori partition at chance level. Reliable content effects existed (seven indices reached ρ≥0.80 at the clip level), but they did not organize along the categories the production team had defined.

### 4.2. Interpretation and Relation to Prior Work

The channel ordering we observe follows directly from response bandwidth. The electrodermal response has a latency of one to five seconds and a recovery of several more [14,15], so a ten-second clip affords at most one or two resolvable responses, and indeed, the majority of trials contained none. Time-domain pulse rate variability requires far longer windows than any clip in this set for stable estimation [16,17], which explains both the negligible stimulus variance and the near-total dominance of between-person variance in pulse rate (93.5%), a channel that in effect measures who the participant is rather than what they are watching. Oculomotor and facial channels, sampling at 60 and 30 Hz respectively, accumulate hundreds of observations per clip and correspondingly resolve content.

This interpretation is consistent with, but sharper than, the mixed picture in the applied literature. Studies reporting successful autonomic discrimination of media content typically use exposures of one to several minutes [7,8], where the electrodermal and cardiac systems have time to express content differences; studies at advertisement length more often report weak or inconsistent autonomic effects, a pattern that our variance decomposition explains quantitatively rather than attributing to sample size alone. The reliability results connect this literature to the broader reliability crisis documented for individual-difference measures derived from robust experimental effects [20,21]: an index can produce a highly significant group-level effect and still be nearly useless for ranking stimuli, because significance and reliability depend on different variance ratios.

The inter-subject correlation result qualifies the negative electrodermal finding in an important way. Tonic electrodermal trajectories were reliably time-locked in 18 of 20 clips, with a median ISC of 0.446, comparable in magnitude to values reported for neural and physiological synchrony measures that predict audience-level outcomes [38,39,40]. Skin conductance therefore does respond to this material; what fails is the interval-averaged summary. That ISC correlated negativelywith SCR rate (ρ=−0.537) reinforces the point: the two quantities are not noisy versions of a single construct. For short-form content, the practical implication is that electrodermal activity should be analyzed as a time series aligned across viewers, not as a scalar arousal score.

The time-resolved comparison supports the response-bandwidth account more directly than the scalar analysis alone. Pupil diameter, an autonomic channel with a latency of a few hundred milliseconds, was reliable both as a clip mean and as a time course, which shows that autonomic channels are not uninformative at these durations as a class; what fails is a slowly estimated quantity such as a mean pulse rate or RMSSD over a few cardiac cycles. Tonic skin conductance occupies a middle position: its dynamics are reliable, but roughly half of that reliability reflects a shape shared by all clips, and the clip-specific remainder ($\rho_{50}=0.55$) is closer to that of the scalar indices. Facial expression shows the opposite pattern. Its clip means are highly reliable because a minority of expressive viewers smile at the same clips, but in almost half of the trials the signal never leaves the engine floor, so there is little within-clip time course to align. Because the pupil results cannot be separated from luminance here, the pupil should be read as evidence that fast channels are stimulus-locked, not that they index affect [30,31].

The null result for the a priori blocks is informative in itself. It is exactly the outcome one should expect when categories are defined by production-side semantics rather than by response-side properties, and it illustrates why stimulus must be modeled as a random factor: had we analyzed the 1000 trials as independent observations, the effective sample size for a block contrast would have been overstated fiftyfold and several of the non-significant effects in Table 4 would have been reported as robust [22,23,24]. The one surviving effect, higher blink rate for blocks B and C, is interesting in its own right, since blink rate is inversely related to visual attentional demand and is modulated by content pacing [41]; it should nonetheless be treated as a hypothesis for confirmatory testing rather than an established difference, given a stimulus-level sample of five clips per block.

It is also worth separating measurability from validity. The most reliable scalar indices in this study come from facial expression, the channel under the greatest voluntary control. A clip-level engagement score that replicates across viewers could reflect display rules or deliberate social signaling as much as internal state [42]. Reliability shows only that an index measures something about the clip consistently; whether that something is the construct of interest can be decided only against criterion outcomes such as recall, attitude, or choice, which were not collected here. The reliability rankings reported above are therefore a necessary condition for applied use, not evidence of validity.

Finally, the within-session habituation pattern (declining facial engagement, valence, and electrodermal response alongside rising pulse rate) is consistent with combined novelty habituation and postural/attentional fatigue. Because presentation order was fully randomized, this effect is orthogonal to content and would otherwise have been silently absorbed into stimulus estimates. Studies using fixed presentation orders should regard this as a confound of first order.

### 4.3. Implications for Sensor Selection and Study Design

Three concrete recommendations follow. On sensor selection: For exposures below roughly twenty seconds, camera-based facial analysis and eye tracking are the channels that carry recoverable content information, and the fusion of the two outperformed every alternative configuration tested. Electrodermal and photoplethysmographic sensors should be included when the research question concerns individual differences, time-resolved synchrony, or longer exposures, not when the goal is to rank short clips by aggregate arousal. If the goal is to track the moment-to-moment course of a response within a clip, the recommendation changes: tonic electrodermal activity and pupil diameter provide the most reliable time courses (with luminance controlled or measured for the pupil), whereas facial time courses are too sparse to be aligned reliably with 50 viewers. On sample size: Figure 4 provides a directly usable planning tool. A panel of 12–20 viewers suffices for reliable clip-level facial engagement or valence, and 37–44 for gaze dispersion, saccade amplitude, blink rate, or scanpath rate, and no realistic panel suffices for RMSSD at this exposure length. On analysis: Stimulus must enter as a random factor, presentation order must be randomized and modeled, and decoding claims must use participant-wise cross-validation with permutation nulls, since random splitting in a within-subjects design confounds stimulus identity with participant identity [36,37].

### 4.4. Limitations

Several limitations bound these conclusions. The sample is a homogeneous group of young adults (20.4±1.4 years) from a single institution, which maximizes internal validity but limits generalization to older or more heterogeneous audiences; because between-person variance dominates most channels, a more heterogeneous sample would likely lower, not raise, the stimulus variance partition coefficients. The vendor algorithms that produce SCR detections, pulse peaks, fixations, and action-unit scores are treated here as the measurement instrument; alternative pipelines could shift absolute values, although the relative ordering of channels follows from response bandwidth and should be robust. Cardiac activity was recorded photoplethysmographically rather than electrocardiographically, and PPG-derived variability is noisier than its electrocardiographic counterpart [25,26]; a true electrocardiogram would raise the reliability of RMSSD somewhat, but not by the order of magnitude that the present estimates would require. Fifteen participants had Poor gaze calibration, though excluding them did not alter the conclusions. Twenty clips is a modest stimulus-level sample, which limits power for the block contrasts specifically; the negative H3 result should therefore be read as an absence of a large categorical effect rather than as proof of exact null. No self-report, recall, brand attitude, or behavioral outcome was collected, so we cannot evaluate the criterion validity of the reliable indices against downstream advertising effectiveness, which remains the decisive test for applied use [3]. Finally, the semantic characterization of the four content blocks is defined by the production team; response-side categorization of the corpus, which our clustering suggests would differ substantially, is left to future work.

The clip durations (5.57–19.33 s) deserve comment as a limit on generalization. In a data-donation study of TikTok viewing, the interquartile range of the durations of videos actually watched was roughly 10–35 s, only 45% of views were watched to completion, and about a quarter were abandoned before 20% of the video had played [43]. Our clips therefore sample the shorter half of the platform distribution, and because they were watched to completion under laboratory conditions, they approximate the effective exposure of a typical swipe-limited view rather than the full length of a typical upload. Current platforms also allow uploads of several minutes, and the longer end of short-form content is not represented here. The response-bandwidth argument predicts that the autonomic channels should become more competitive as exposure lengthens, so the present estimates are best viewed as conservative for those channels and as specific to the short end of the format.

### 4.5. Future Work

Three extensions follow naturally. First, replicating the variance-partition and reliability analysis across a graded range of exposure durations (10, 30, 60, and 180 s) would map the crossover point at which autonomic channels become competitive, converting the qualitative bandwidth argument into a calibrated design curve. Second, the univariate time-resolved analysis reported here should be extended to joint multivariate models of the pupillary, electrodermal, oculomotor, and facial time series, which are a natural target for the sensor-fusion architectures now common in wearable affective computing [5,9]. The most direct next step, however, is a per-second annotation of clip content (editing cuts, presence and size of the creator’s face on screen, product reveals, text overlays, and audio events), together with frame-wise luminance. Regressing the group time courses of Figure 10 on these annotations would turn the synchrony findings into an explanation of what drives them, separate the common onset response from event-locked responses in electrodermal activity, and remove the luminance confound from the pupil. This coding requires frame-accurate manual annotation of each clip and was beyond the scope of the present analysis. Third, linking the reliable clip-level indices to memory, attitude, and choice outcomes would establish whether the channels that are most measurable are also the most predictive, which is not guaranteed.

## 5. Conclusions

We characterized where stimulus-driven information resides in a five-channel multimodal sensing stack during exposure to short-form influencer videos, using 1000 fully crossed participant × clip trials from 50 viewers and 20 clips. Stimulus-driven variance was strongly channel-specific, reaching 13.7% for fixation rate and 10.2% for facial engagement but only 0.11% for pulse rate and 0.23% for RMSSD. Clip-level estimates were measurement-grade for seven facial and oculomotor indices at a panel size of 50 and would require 134 to 370 viewers for the autonomic indices. The modalities were nearly orthogonal, with between-modality dependence 5.9 times weaker than within-modality dependence and five components retained by parallel analysis, and their fusion identified individual clips at 16.8% balanced accuracy against a 5% chance level, though only when the uninformative autonomic channels were excluded. Electrodermal activity was nonetheless reliably time-locked across viewers in 18 of 20 clips, showing that its failure in aggregate analyses reflects the summary statistic rather than the sensor. A per-second comparison of nine channels confirmed this point more generally: tonic electrodermal activity and pupil diameter gave the most reliable time courses, facial time courses were sparse and less reliable, and pulse rate was unreliable under both treatments. The four a priori content blocks explained essentially nothing beyond the identity of individual clips.

For the design of multimodal engagement studies at short exposure durations, the practical conclusions are that camera-based facial and oculomotor sensing should be prioritized, that electrodermal activity should be analyzed as a synchronized time series rather than as a scalar, that panel sizes should be set per channel from the reliability curves reported here, and that stimulus identity, presentation order, and participant identity must all enter the inferential model explicitly.

## Figures and Tables

**Table 1:** Descriptive statistics of the twelve primary indices over the full corpus and by content block (mean ± standard deviation of untransformed values). *n* is the number of analyzable trials after quality control.

Index (Unit)	Modality	*n*	Overall	Block A	Block B	Block C	Block D	Median
SCR rate (min^−1^)	EDA	863	3.16±4.29	3.17±4.21	2.95±4.05	3.14±4.17	3.39±4.71	0.00
SCL drift (μS s^−1^)	EDA	863	0.000±0.020	0.00±0.02	−0.00±0.02	−0.00±0.01	0.00±0.02	−0.001
Pulse rate (bpm)	PPG	942	78.09±13.28	78.41±13.53	78.06±13.38	78.26±13.24	77.64±13.04	77.24
log RMSSD (log ms)	PPG	942	4.20±0.61	4.19±0.63	4.18±0.62	4.20±0.58	4.22±0.62	4.18
Blink rate (min^−1^)	OCU	1000	18.60±15.36	16.20±14.88	20.83±16.00	20.86±15.10	16.51±14.86	15.63
Fixation rate (min^−1^)	OCU	1000	102.60±35.34	97.16±37.00	104.78±34.62	98.57±28.78	109.88±38.85	102.56
Median fixation duration (ms)	OCU	1000	385.6±293.2	420.1±333.9	389.7±376.6	356.2±185.8	376.3±234.2	319.81
Gaze dispersion (px)	OCU	1000	152.9±56.0	162.9±66.4	140.9±48.8	156.0±49.5	151.8±55.5	149.03
Saccade amplitude (deg)	OCU	1000	3.45±1.13	3.76±1.38	3.30±1.14	3.37±0.90	3.38±0.99	3.39
Scanpath rate (deg s^−1^)	OCU	1000	5.35±2.87	5.41±3.11	5.18±2.79	5.31±2.76	5.50±2.80	4.97
Facial engagement (%)	FEA	996	10.73±22.46	17.78±29.37	6.90±17.12	7.13±16.54	11.06±22.67	0.34
Facial valence (a.u.)	FEA	996	9.02±20.59	15.41±26.88	5.90±15.81	5.87±15.06	8.87±20.93	0.00

EDA, electrodermal activity; PPG, photoplethysmography; OCU, oculomotor; FEA, facial expression analysis; SCR, skin conductance response; SCL, skin conductance level.

**Table 2:** Stimulus inventory and clip-level response profile. Values are means of person-centered rank-based normal scores across the 50 viewers of each clip; ISC is the leave-one-out inter-subject correlation of tonic electrodermal activity.

Clip	Block	Duration (s)	SCR Rate	Pulse Rate	Blink Rate	Fixation Rate	Facial Engag.	Facial Valence	ISC
A11	A	10.35	−0.16	0.02	−0.33	0.58	0.29	0.19	0.35
A2_s	A	9.73	−0.17	−0.07	−0.40	−0.19	0.45	0.43	0.31
A3_s-1	A	14.41	0.22	0.16	−0.13	−0.14	0.76	0.83	0.30
A5	A	8.79	0.09	−0.02	−0.03	−0.67	−0.28	−0.26	0.42
A8_s	A	9.54	0.00	−0.02	−0.04	−0.34	−0.15	−0.10	0.56
B10	B	8.24	0.06	0.03	0.05	−0.22	−0.06	0.05	0.20
B2	B	9.37	0.00	−0.01	0.30	0.09	−0.03	−0.12	0.71
B3	B	11.68	−0.08	0.02	0.14	0.49	−0.11	−0.08	0.47
B5	B	11.06	−0.14	−0.03	0.17	0.18	−0.27	−0.16	0.68
B7	B	19.12	−0.04	−0.03	0.17	−0.22	−0.22	−0.24	0.61
C10	C	14.63	−0.08	0.02	0.23	−0.06	−0.01	0.10	0.68
C2	C	17.41	0.08	−0.01	0.22	−0.13	0.11	−0.03	0.54
C3	C	19.27	−0.05	−0.01	0.18	−0.06	−0.12	−0.10	0.21
C6	C	17.52	0.08	0.04	0.26	−0.31	−0.11	−0.16	0.10
C9	C	15.01	0.02	0.05	0.01	−0.01	−0.05	−0.18	0.50
D10	D	13.29	−0.08	−0.05	−0.07	0.16	−0.19	−0.20	0.58
D11	D	11.48	0.06	−0.04	−0.12	0.04	−0.34	−0.12	0.30
D12	D	15.15	0.19	−0.01	−0.36	0.74	0.47	0.38	0.24
D8	D	12.85	0.16	−0.06	−0.01	−0.58	−0.04	−0.16	0.18
D9	D	5.73	−0.19	0.04	−0.25	0.66	−0.10	−0.08	0.65

**Table 3:** Variance partition and clip-level reliability of the twelve primary indices and of one supplementary index. VPC, variance partition coefficient (%); LRT, likelihood-ratio statistic for $\sigma_{v}^{2}=0$; *q*, Benjamini–Hochberg-adjusted *p*-value across the twelve indices; $r_{1/2}$, median split-half correlation of clip means over 1000 participant partitions with 95% resampling interval; $\rho_{50}$, Spearman–Brown projection to the full sample; $k^{*}$, viewers required for ρ=0.80.

Index	Mod.	VPC_part_	VPC_stim_	VPC_res_	LRT	*q*	$r_{1/2}$ [95% RI]	$\rho_{50}$ ($k^{*}$)
SCR rate	EDA	51.82	1.01	47.17	4.89	0.016	0.34 [0.03, 0.62]	0.51 (180)
SCL drift	EDA	6.18	2.57	91.26	7.21	0.005	0.41 [0.14, 0.69]	0.58 (134)
Pulse rate	PPG	93.52	0.11	6.37	3.97	0.025	0.35 [−0.03, 0.65]	0.52 (179)
log RMSSD	PPG	74.12	0.23	25.65	1.33	0.125	0.21 [−0.09, 0.52]	0.34 (370)
Blink rate	OCU	54.81	3.93	41.26	52.93	<0.001	0.73 [0.53, 0.86]	0.84 (38)
Fixation rate	OCU	33.63	13.65	52.72	168.03	<0.001	0.88 [0.78, 0.94]	0.94 (14)
Median fixation duration	OCU	39.34	1.10	59.55	4.93	0.016	0.35 [0.03, 0.63]	0.52 (186)
Gaze dispersion	OCU	22.25	6.95	70.81	54.65	<0.001	0.73 [0.55, 0.86]	0.85 (37)
Saccade amplitude	OCU	37.74	5.31	56.94	51.52	<0.001	0.73 [0.55, 0.86]	0.85 (37)
Scanpath rate	OCU	48.58	3.96	47.46	44.66	<0.001	0.69 [0.50, 0.85]	0.82 (44)
Facial engagement	FEA	50.47	10.15	39.37	167.44	<0.001	0.89 [0.80, 0.94]	0.94 (12)
Facial valence	FEA	50.77	7.22	42.01	107.24	<0.001	0.84 [0.73, 0.91]	0.91 (19)
Supplementary index (not in the primary family; *p* uncorrected)								
Pupil diameter	PUP	85.84	3.83	10.33	242.72	<0.001	0.91 [0.82, 0.96]	0.95 (9)

**Table 4:** Tests of the a priori content structure. Mixed-model results are likelihood-ratio tests of the block factor in crossed random-effects models controlling for clip duration and presentation position; permutation results reassign block labels across the twenty clip means. Block means are in person-centered normal-score units.

Index	LRT(3)	*q*_LMM_	$F_{\mathrm{perm}}$	*q*_perm_	A	B	C	D
SCR rate	1.90	0.791	0.25	0.903	0.07	0.04	0.07	0.11
SCL drift	4.83	0.444	1.05	0.795	0.09	−0.14	−0.02	0.07
Pulse rate	2.15	0.791	0.68	0.903	0.02	−0.00	0.02	−0.04
log RMSSD	3.35	0.681	0.38	0.903	−0.02	−0.02	0.01	0.03
Blink rate	19.19	**0.003**	12.06	**0.005**	−0.17	0.18	0.19	−0.15
Fixation rate	2.84	0.714	0.89	0.795	−0.15	0.06	−0.11	0.20
Median fixation duration	1.10	0.931	0.43	0.903	0.06	−0.01	−0.05	−0.00
Gaze dispersion	<0.01	1.000	1.51	0.590	0.15	−0.20	0.07	−0.02
Saccade amplitude	7.39	0.346	2.76	0.339	0.24	−0.15	−0.05	−0.04
Scanpath rate	0.70	0.954	0.20	0.903	0.01	−0.06	−0.00	0.05
Facial engagement	6.09	0.346	1.53	0.590	0.27	−0.08	0.03	0.02
Facial valence	5.93	0.346	1.63	0.590	0.23	−0.09	−0.06	−0.02
Multivariate: PERMANOVA on clip profiles, F=1.30, $R^{2}=0.196$, p=0.175 (9999 permutations)								
Cluster agreement: adjusted Rand index =0.075, p=0.148 (9999 permutations)								

Values in bold are significant after false-discovery-rate correction (q<0.05).

**Table 5:** Multimodal decoding of clip identity (20 classes, chance =5%) under participant-wise nested cross-validation. Null distributions were obtained by permuting labels within participants and repeating the full cross-validation. The pulse channel is photoplethysmographic; no electrocardiogram was recorded.

Feature Set	Balanced Accuracy (%)	Top-5 Accuracy (%)	Null (Mean ± SD, %)	*p*
Electrodermal (2)	5.9	28.5	4.98±0.55	0.079
Pulse, PPG (2)	6.1	28.4	4.94±0.64	0.059
Facial (2)	8.5	35.5	5.10±0.64	0.010
Electrodermal + pulse, PPG (4)	7.7	28.5	5.04±0.68	0.010
Oculomotor (6)	15.1	48.5	5.00±0.77	0.010
Oculomotor + facial (8)	**16.8**	**52.7**	4.89±0.64	0.010
All modalities (12)	16.2	51.4	4.96±0.71	0.010
All modalities, duration-residualized (12)	15.1	49.4	4.97±0.83	0.010

Bold values indicate the best-performing feature set.

**Table 6:** Time-resolved comparison of nine per-second channels. Flat, trials whose series was constant over the whole clip; ISC, leave-one-out inter-subject correlation of within-trial standardized series (median and interquartile range across the twenty clips); sig., clips significant after false-discovery-rate control; κ, median Fleiss’ κ for per-second responding labels; $r_{1/2}$, median split-half correlation of concatenated group time courses with 95% resampling interval; $\rho_{50}$ ($k^{*}$), Spearman–Brown projection to 50 viewers and viewers required for ρ=0.80; shape removed, $\rho_{50}$ after subtraction of the across-clip template.

Channel	Mod.	Flat (%)	ISC [IQR]	Sig.	κ (Sig.)	$r_{1/2}$ [95% RI]	$\rho_{50}$ ($k^{*}$)	Shape Removed
Tonic skin conductance	EDA	0.0	0.53 [0.33, 0.66]	18	0.100 (17)	0.76 [0.66, 0.82]	0.88 (27)	0.55
Pulse rate	PPG	0.0	0.03 [0.00, 0.12]	1	0.003 (4)	0.15 [0.05, 0.24]	0.28 (519)	0.21
Pupil diameter	PUP	0.0	0.48 [0.34, 0.61]	20	0.090 (18)	0.86 [0.83, 0.89]	0.93 (16)	0.90
Gaze velocity	OCU	0.0	0.16 [0.13, 0.22]	17	0.018 (8)	0.54 [0.48, 0.60]	0.70 (84)	0.68
Blink count	OCU	10.6	0.12 [0.07, 0.21]	8	0.013 (8)	0.46 [0.38, 0.53]	0.64 (111)	0.57
Facial engagement	FEA	44.1	0.08 [0.03, 0.18]	6	0.009 (4)	0.38 [0.28, 0.46]	0.69 (89)	0.69
Facial valence	FEA	46.7	0.18 [0.04, 0.23]	6	0.016 (7)	0.36 [0.24, 0.46]	0.68 (94)	0.69
AFFDEX joy	FEA	0.0	0.11 [0.07, 0.19]	6	0.010 (5)	0.44 [0.34, 0.52]	0.61 (127)	0.60
AFFDEX surprise	FEA	0.0	0.06 [0.03, 0.12]	1	0.006 (1)	0.20 [0.10, 0.30]	0.34 (392)	0.23

EDA, electrodermal activity; PPG, photoplethysmography; PUP, pupillometry; OCU, oculomotor; FEA, facial expression analysis. For facial engagement and valence, the ISC and reliability are computed on the non-flat series only.

## Data Availability

The derived, fully anonymized trial-level dataset and the analysis code supporting the reported results are available from the corresponding author upon reasonable request. Raw sensor exports and facial video are not available because they contain personally identifying information.

## Abbreviations

The following abbreviations are used in this manuscript:

ARI	Adjusted Rand index
AU	Action unit
EDA	Electrodermal activity
FDR	False discovery rate
FEA	Facial expression analysis
HRV	Heart rate variability
IBI	Interbeat interval
ISC	Inter-subject correlation
I-VT	Velocity-threshold identification
LRT	Likelihood-ratio test
OCU	Oculomotor
PERMANOVA	Permutational multivariate analysis of variance
POF	Polymer optical fiber
PPG	Photoplethysmography
PUP	Pupillometry (pupil diameter)
RMSSD	Root mean square of successive differences
SCL	Skin conductance level
SCR	Skin conductance response
SNR	Signal-to-noise ratio
VPC	Variance partition coefficient

## Appendix A. Covariate and Robustness Analyses

All appendix analyses use the same crossed random-effects specification as the main text, with participant and stimulus as crossed random intercepts and rank-based normal scores as outcomes.

**Table A1:** Effects of participant sex, participant age, presentation position, and clip duration on the primary indices. Coefficients are in normal-score units; *q* values are Benjamini–Hochberg-adjusted within each covariate family across the twelve indices.

Index	$\beta_{\mathrm{sex}}$	*q*	$\beta_{\mathrm{age}}$	*q*	$\beta_{\mathrm{order}}$	*q*	$\beta_{\mathrm{dur}}$	*q*
SCR rate	0.444	0.091	−0.006	0.924	−0.060	**0.004**	0.034	0.567
SCL drift	0.141	0.395	0.070	0.484	−0.068	0.060	0.020	0.889
Pulse rate	−0.313	0.400	0.091	0.854	0.049	**<0.001**	0.004	0.889
log RMSSD	−0.008	0.976	−0.009	0.924	−0.082	**<0.001**	0.037	0.304
Blink rate	−0.226	0.400	−0.037	0.907	0.019	0.431	0.085	0.304
Fixation rate	0.216	0.395	−0.012	0.924	0.020	0.431	−0.056	0.889
Median fixation duration	−0.350	0.277	0.038	0.907	−0.045	0.098	−0.021	0.889
Gaze dispersion	−0.037	0.860	0.083	0.535	0.023	0.431	−0.010	0.889
Saccade amplitude	−0.311	0.279	0.072	0.728	0.012	0.613	−0.078	0.567
Scanpath rate	0.090	0.783	0.023	0.924	0.051	**0.039**	−0.011	0.889
Facial engagement	0.233	0.395	0.070	0.728	−0.071	**<0.001**	0.017	0.889
Facial valence	−0.115	0.736	−0.050	0.907	−0.093	**<0.001**	−0.008	0.889

$\beta_{\mathrm{sex}}$ is the contrast of male minus female. Positive $\beta_{\mathrm{order}}$ indicates an increase across the session. Values in bold are significant after false-discovery-rate correction (q<0.05).

**Table A2:** Robustness of the stimulus variance partition to eye-tracker calibration quality. The right-hand column recomputes ${\mathrm{VPC}}_{\mathrm{stim}}$ after excluding the fifteen participants whose calibration was graded Poor.

Index	VPC_stim_, Full Sample (%)	VPC_stim_, Poor Excluded (%)	Δ (pp)
SCR rate	1.01	1.13	+0.12
SCL drift	2.57	2.36	−0.20
Pulse rate	0.11	0.18	+0.07
log RMSSD	0.23	0.08	−0.15
Blink rate	3.93	3.07	−0.86
Fixation rate	13.65	17.13	+3.48
Median fixation duration	1.10	1.25	+0.15
Gaze dispersion	6.95	8.02	+1.07
Saccade amplitude	5.31	7.45	+2.14
Scanpath rate	3.96	4.89	+0.94
Facial engagement	10.15	10.70	+0.54
Facial valence	7.22	7.04	−0.18

## Appendix B. Component Structure

Table A3 reports the loadings of the twelve primary indices on the first four principal components of the person-centered correlation matrix, together with the eigenvalues and the parallel-analysis thresholds used to determine the number of retained components. Five components exceeded their parallel-analysis threshold; the fifth is not tabulated because no index loaded above |0.45| on it.

**Table A3:** Principal component loadings, eigenvalues, and parallel-analysis thresholds.

Index	PC1	PC2	PC3	PC4
SCR rate	−0.06	−0.37	−0.21	−0.46
SCL drift	−0.00	−0.56	−0.16	−0.40
Pulse rate	0.12	−0.30	0.03	0.52
log RMSSD	−0.08	−0.14	−0.08	−0.66
Blink rate	0.29	0.10	0.13	−0.15
Fixation rate	0.72	−0.12	0.53	−0.08
Median fixation duration	−0.74	0.12	−0.36	0.14
Gaze dispersion	0.68	0.16	−0.43	0.05
Saccade amplitude	0.43	0.22	−0.80	0.06
Scanpath rate	0.92	0.02	−0.08	−0.01
Facial engagement	0.12	−0.81	−0.06	0.18
Facial valence	−0.02	−0.76	−0.16	0.28
Eigenvalue	2.656	1.905	1.361	1.243
Parallel-analysis 95th percentile	1.246	1.179	1.133	1.098
Variance explained (%)	22.1	15.9	11.3	10.4
